# Supplementary material for: Unusual nuclear exchange within a germanium-containing aromatic ring that results in germanium atom transfer
Source: Nat Commun. 2023 Jul 28;14:4519. doi: 10.1038/s41467-023-40188-y (PMC10382490; doi:10.1038/s41467-023-40188-y)
Supplement: Supplementary file 1 — Supplementary Information [file 41467_2023_40188_MOESM1_ESM.pdf]

Supplementary Information

Unusual Nuclear Exchange within a Germanium-containing Aromatic Ring that Results in Germanium Atom Transfer

Ryohei Nishino[1], Norihiro Tokitoh[1,2]*, Ryuto Sasayama[1], Rory Waterman[3], and Yoshiyuki Mizuhata[1,2]*

Affiliations:

[1] Institute for Chemical Research, Kyoto University, Gokasho, Uji, Kyoto 611-0011, Japan.

[2] Integrated Research Consortium on Chemical Sciences, Gokasho, Uji, Kyoto 611-0011, Japan.

[3] Department of Chemistry, University of Vermont, Burlington, Vermont 05405-0125, USA.

*Correspondence to: mizu@boc.kuicr.kyoto-u.ac.jp (YM), tokitoh@boc.kuicr.kyoto-u.ac.jp (NT)

Contents

1. Materials and methods 2

2. Experimental procedures for preparation and reaction of new compounds 3

3. X-Ray crystallographic analysis 7

4. NMR spectra of new compounds 16

5. NMR spectra of the reaction products in the mechanistic investigation 26

6. Computational details 36

7. References 56

1. Materials and methods

All reactions were carried out under argon atmosphere using standard Schlenk techniques or glovebox (Yamato YGB1-CS). All reaction solvents were purified by Ultimate Solvent System (Glass Contour Company)[1] while all deuterated solvents were dried over Molecular sieves 4A before use. (1*E*,2*E*)-N^1,N^2-bis(2,6-diisopropylphenyl)ethane-1,2-diimine (diimine),[2] 1,3-diisopropyl-4,5-dimethyl-imidazol-2-ylidene ($Im^{iPr2Me2}$),[3] 1-Tbb-2-*tert*-butyl-1-chlorogermacyclohexa-2,5-diene (Tbb = 4-*tert*-butyl-2,6-bis[bis(trimethylsilyl)methyl]phenyl),[4] potassium 2-*tert*-butylgermabenzenide **1**,[4] 1,2-bis(Tbb)-1,2-dibromodigermene **2-Ge**,[5] and 1,2-bis(Tbb)-1,2-dibromodisilene **2-Si**[6] were synthesized according to literature procedures. Solid lithium diisopropylamide was synthesized by the reaction of 1 equivalent of *n*-butyllithium and diisopropylamine in diethyl ether and stored in the glovebox.

^{1}H and ^{13}C NMR spectra were measured on Bruker Avance NEO 400, Avance NEO 600, or JEOL ECS-400 NMR spectrometer. ^{1}H NMR chemical shifts in C_6D_6 were referenced to the residual peak of the solvent; C_6D_5H (δ 7.15). ^{13}C NMR chemical shifts in C_6D_6 were referenced to the peak of solvent; C_6D_6 (δ 128.0). The multiplicity of the signals in the ^{13}C NMR spectra was determined by DEPT techniques. All signals of each compound were assigned using various 2D-NMR (COSY, HSQC, HMBC, NOESY, and ROESY) techniques. High-resolution mass spectrometry data were obtained on Bruker Daltonics micrOTOF focus-Kci with IonSence DART®-SVP ion source. Melting points were measured by BUCHI M-565 apparatus and thermometer was uncorrected. Elemental analyses were carried out at Microanalytical Laboratory of Institute for Chemical Research, Kyoto University.

2. Experimental procedures for preparation and reaction of new compounds

Preparation of 1-Tbb-2-*tert*-butylgermabenzene 3-Ge.

To a hexane (30 mL) solution of 1-Tbb-2-*tert*-butyl-1-chlorogermacyclohexa-2,5-diene (1.09 g, 1.60 mmol), a THF (2 mL) solution of lithium diisopropylamide (0.207 g, 1.93 mmol) was added at room temperature and stirred for 1 h. After the solvent was exchanged to hexane, inorganic salts were removed by Celite® filtration. Removal of the solvents and purification by sublimation gave Tbb-germabenzene **3-Ge** (0.353 g, 0.548 mmol, 34%) as colorless solid: m.p. 73–76 °C; Anal. Calcd. for $C_{33}H_{62}GeSi_4$: C, 61.56; H, 9.71. Found: C, 61.47, H, 9.81; HRMS (*m*/*z*) $[M+H]^+$: calcd. for $C_{33}H_{63}Si_4Ge$, 645.3220; found, 645.3222; ^1H NMR (600 MHz, C_6D_6): δ 0.11 (s, 18H), 0.16 (s, 18H), 1.32 (s, 9H), 1.48 (s, 9H), 2.29 (s, 2H), 6.70-6.78 (m, 1H), 6.98 (s, 2H), 7.95-8.05 (m, 3H); ^{13}C NMR (150 MHz, C_6D_6): δ 0.9 (q), 1.7 (q), 31.2 (q), 34.0 (q), 34.4 (d), 34.7 (s), 38.5 (s), 114.5 (d), 122.0 (d), 130.1 (s), 138.0 (d), 138.3 (d), 139.2 (d), 149.0 (s), 151.6 (s), 162.9 (s).

Independent preparation of potassium Tbb[bis(2-*tert*-butylgermabenzenyl)]germylide 4 by the reaction of potassium 2-*tert*-butylgermabenzenide 1 with 0.25 eq. of 1,2-bis(Tbb)-1,2-dibromodigermene 2-Ge.

To a THF (5 mL) solution of potassium germabenzenide **1** (46.6 mg, 0.200 mmol), a THF (5 mL) solution of 1,2-dibromodigermene **2-Ge** (60.1 mg, 0.0499 mmol) was added at –40 °C, and stirred for 1 h at room temperature. After the solvent was exchanged to benzene, the inorganic salts were removed by filtration. Removal of the solvents *in vacuo* and washing with hexane gave **4** (67.6 mg, 0.0712 mmol, 71%) as orange solid: m.p. 213 °C (dec.); HRMS (*m*/*z*) $[M–K+O_2+H_2O]^-$: calcd. for $C_{42}H_{77}O_3Si_4Ge_3$, 959.2625; found, 959.2585; ^1H NMR (600 MHz, C_6D_6): δ 0.32 (s, 36H), 1.47 (s, 9H), 1.61 (s, 18H) 3.10 (s, 2H), 6.14 (ddd, 2H, J = 8.4, 7.8 Hz, 1.2 Hz), 7.03 (s, 2H), 7.24 (ddd, 2H, J = 10.8, 7.8, 1.2 Hz), 7.47 (dd, 2H, J = 8.4, 1.2 Hz), 8.35 (dd, 2H, J = 10.8, 1.2 Hz); ^{13}C NMR (150 MHz, C_6D_6): δ 1.7 (q), 31.7 (q), 34.2 (s), 34.3 (d), 34.5 (q), 39.8 (s), 111.7 (d), 122.0 (d), 133.0 (d), 134.2 (d), 146.4 (s), 148.7 (s), 149.0 (s), 158.2 (d), 179.2 (s).

Reaction of potassium Tbb[bis(2-*tert*-butylgermabenzenyl)]germylide 4 with 1,2-bis(Tbb)-1,2-dibromodigermene 2-Ge.

To a mixture of **4** (23.8 mg, 0.0250 mmol) and **2-Ge** (15.1 mg, 0.0125 mmol), C_6D_6 (1 mL) and 1,4-dioxane (5.0 μL, 0.0585 μmol, as an internal standard for determining yields by NMR) were added. The color of the solution was immediately changed to brownish orange. Heating the mixture at 75 °C overnight and subsequent exposure to room light for 3 h gave the mixture of **3-Ge** and **5-2** by the following yields based on the ^{1}H NMR; **3-Ge**: 41%; **5-2**: 57%. After the inorganic salts and solvents were removed by filtration and vacuum, recrystallization from hexane gave **5-2** (8.5 mg, 0.0055 mmol, 22%) as blue green crystals.

Isolation of Ge/C cluster 5-1 by the reaction of potassium Tbb[bis(2-*tert*-butylgermabenzenyl)]germylide 4 with 1,2-bis(Tbb)-1,2-dibromodigermene 2-Ge.

To a benzene (3 mL) solution of **4** (71.4 mg, 0.0752 mmol), benzene (3 mL) solution of **2-Ge** (45.3 mg, 0.0376 mmol) was added at room temperature and stirred at 75 °C for 12 h in the dark. After the inorganic salts were removed by filtration, solvents were removed *in vacuo*. Recrystallization from hexane in the dark gave red crystals of **5-1** (8.6 mg, 0.0057 mmol, 11%); HRMS (DART): Calcd. for $C_{66}H_{125}Ge_5Si_8$ $[M+H]^+$: 1505.4055; found: 1505.4066 $[M+H]^+$; Anal. Calcd. for $C_{66}H_{124}Ge_5Si_8$: C, 52.65; H, 8.30; Found: C, 52.72; H, 8.49; ^{1}H NMR (600 MHz, C_6D_6): δ 0.14 (s, 9H), 0.17 (s, 18H), 0.327 (s, 9H), 0.331 (s, 9H), 0.36 (s, 18H), 0.44 (s, 9H), 1.20 (s, 9H), 1.31 (s, 9H), 1.32 (s, 9H), 1.39 (s, 9H), 1.93 (s, 2H), 2.08 (s, 1H), 2.23 (d, 1H, J = 7.2 Hz), 3.33 (s, 1H), 3.39 (dd, 1H, J = 9.6, 3.0 Hz), 4.95 (dd, 1H, J = 8.4, 7.2 Hz), 5.75 (ddd, 1H, J = 10.2, 7.8, 3.0 Hz), 6.25-6.30 (m, 2H), 6.45 (d, 1H, J = 7.8 Hz), 6.55 (d, 1H, J = 10.8 Hz), 6.88-6.90 (m, 4H); ^{13}C NMR (150 MHz, C_6D_6): δ 1.3 (q), 1.4 (q), 1.95 (q), 2.02 (q), 2.6 (q), 3.4 (q), 25.7 (d), 30.9 (d), 31.2 (q), 31.3 (q), 32.4 (d), 32.6 (q), 32.7 (d), 33.1 (d), 34.27 (s), 34.32 (s), 35.8 (q), 37.0 (s), 38.2 (d), 39.6 (s), 122.0 (d), 122.1 (d), 123.9 (d), 125.4 (d), 127.6 (d), 128.3 (d), 131.8 (d), 131.5 (d), 137.4 (s), 138.8 (s), 141.0 (s), 148.5 (s), 149.4 (s), 149.5 (s), 150.0 (s), 150.4 (s), 159.2 (s).

Reaction of potassium Tbb[bis(2-*tert*-butylgermabenzenyl)]germylide 4 with 1,2-bis(Tbb)-1,2-dibromodigermene 2-Ge in the presence of 1,3-diisopropyl-4,5-dimethylimidazol-2-ylidene ($Im^{iPr2Me2}$).

To a C_6D_6 (0.5 mL) solution of **2-Ge** (6.0 mg, 0.0050 mmol), $Im^{iPr2Me2}$ (2.5 mg, 0.0139 mmol)

and a C_6D_6 (0.5 mL) solution of **4** (9.5 mg, 0.010 mmol) were added at room temperature. The mixture was transferred to J. Young NMR tube which was put hexamethylbenzene (1.4 mg, 0.0086 mmol, as an internal standard for determining yields by NMR) and heated at 50 °C for 2 days. The ^{1}H NMR spectrum indicated the consumption of **4** (0.0021 mmol, 22% remained) and the formation of **6·NHC** (0.0104 mmol, 52%).

Preparation of Tbb(2-*tert*-butylgermabenzenyl)germylene NHC adduct (6·NHC).

To a benzene (3 mL) solution of **2-Ge** (50.1 mg, 0.0416 mmol), $Im^{iPr2Me2}$ (15.0 mg, 0.0832 mmol) was added at room temperature and stirred for 5 minutes. After the color of the solution was changed from orange to colorless, **1** (20.3 mg, 0.0817 mmol) was added at room temperature and stirred overnight. The inorganic salts and solvents were removed by filtration and vacuum. Washing with hexane gave germabenzenylgermylene NHC adduct **6·NHC** (65.2 mg, 0.0727 mmol, 87%) as yellow solid: m.p. 183 °C (dec.); Anal. Calcd. for $C_{44}H_{82}Ge_2Si_4N_2$: C, 58.93; H, 9.22; N, 3.12. Found: C, 58.97; H, 9.41, N; 3.24; ^{1}H NMR (600 MHz, C_6D_6): δ 0.10 (s, 18H), 0.35 (s, 18H) 0.81 (d, 6H, J = 7.2 Hz), 1.34 (d, 6H, J = 7.2 Hz), 1.39 (s, 9H), 1.57 (s, 6H), 1.84 (s, 9H), 3.18 (brs, 2H), 5.80 (brs, 2H), 6.90 (s, 2H), 7.00 (ddd, 1H, ^1J = 9.0, 8.4, 1.2 Hz), 7.99 (ddd, 1H, J = 10.8, 8.4, 1.2 Hz), 8.12 (dd, 1H, J = 9.0, 1.2 Hz), 8.45 (dd, 1H, J = 10.8, 1.2 Hz); ^{13}C NMR (150 MHz, C_6D_6): δ 2.4 (q), 10.2 (q), 21.9 (q), 22.4 (q), 30.5 (d), 31.6 (q), 34.2 (s), 35.3 (q), 40.3 (s), 53.5 (d), 115.6 (d), 122.8 (d), 126.9 (s), 134.9 (d), 135.4 (d), 147.1 (s), 147.8 (s), 149.0 (s), 154.3 (d), 174.9 (s), 179.7 (s).

Thermolysis of Tbb(2-*tert*-butylgermabenzenyl)germylene NHC adduct (6·NHC).

To **6·NHC** (2.4 mg, 0.0026 mmol) in J. Young NMR tube, C_6D_6 (ca. 1 mL) and hexamethylbenzene (1.3 mg, 0.0080 mmol, internal standard for determining yields by NMR) were added and heated for 1 day at 110 °C. The ^{1}H NMR spectrum indicated the complete consumption of **6·NHC** and the formation of germabenzene **3-Ge** (0.0017 mmol, 66%).

Thermolysis of Tbb(2-*tert*-butylgermabenzenyl)germylene NHC adduct (6·NHC) in the presence of (1*E*,2*E*)-*N*^{1},*N*^{2}-bis(2,6-diisopropylphenyl)ethane-1,2-diimine.

6·NHC (10.1 mg, 0.0113 mmol), diimine (0.0113 mmol), and hexamethylbenzene (2.0 mg, 0.012 mmol, as an internal standard) were put into the pressure-resistant NMR tube and dissolved in C_6D_6 (1 mL). The mixture was heated for 5 days at 110 °C. The ^{1}H NMR spectrum indicated the complete

consumption **6·NHC** and the formation of germabenzene **3-Ge** (0.0079 mmol, 70%), $Im^{iPr2Me2}$ (0.0956 mmol, 78%), and *N*-heterocyclic germylene (NHGe, 0.0045 mmol, 40%). The formation of NHGe was confirmed by the comparison with the reported spectral data.[7]

Reaction of potassium 2-*tert*-butylgermabenzenide 1 with 1.25 eq. of 1,2-bis(Tbb)-1,2-dibromodigermene 2-Ge.

To the hexane solution of **2-Ge** (37.8 mg, 0.0313 mmol), **1** (6.0 mg, 0.026 mmol) was added at room temperature and stirred for 12 h at 60 °C. The ^{1}H NMR spectrum indicated the formation of **3-Ge** and 2,4,5-tribromopentagerma[1.1.1]propellane **7** by the following NMR yields based on 1,4-dioxane (4.0 μL, 0.0467 mmol): **3-Ge**: 0.026 mmol, 99%; **7**: 0.0086 mmol, 66%. After the inorganic salts were removed by filtration, all solvents were removed *in vacuo*. Washing with hexane gave **7** (14.5 mg, 0.00743 mmol, 60%) as red solid: m.p. 190-191 °C; Anal. Calcd. for $C_{78}H_{161}Br_3Ge_5Si_{12}$ (**7**·hexane): C, 45.95; H, 7.96. Found: C, 46.07; H, 7.94; HRMS (*m*/*z*) $[M+H]^+$: calcd. for $C_{72}H_{148}Si_{12}Ge_5Br_3$, 1953.2445; found, 1953.2438; ^{1}H NMR (600 MHz, C_6D_6): δ 0.39 (s, 108H), 1.25 (s, 27H), 3.00 (s, 6H), 6.93 (s, 6H); ^{13}C NMR (150 MHz, C_6D_6): δ 2.49 (q), 31.1 (q), 33.0 (d), 34.4 (s), 123.1 (d), 143.3 (s), 148.2 (s), 151.7 (s).

3. X-Ray crystallographic analysis

All single crystals were obtained by slow evaporation technique using the solvent, and the crystal data were summarized in Supplementary Table 1 and 2.

Crystals of **4** were obtained as the complex with 18-crown-6 ether (18c6) which was prepared by adding equimolar amounts of 18c6 to the benzene solution of **4**. The X-ray diffraction were collected by Bruker-D8 Venture diffractometer. Graphite monochromated Mo *Kα* radiation was used as the X-ray source. Data reductions were performed using Bruker SAINT software. Structures were solved by direct methods (SHELXT)[8] and refined against F^2 by weighted full matrix least-squares (SHELXL).[9]

For the structure refinement of **5-2**, an inverted disorder of whole molecule was observed. Due to the low occupancy of the minor part (solved as 98:2 occupancy ratio), Ge atoms and two silicon atoms on the Tbb group could be assigned.

CheckCIF report of compound **7** gave one alert-B (`PLAT910_ALERT_3_B Missing # of FCF Reflection(s) Below Theta(Min)          14 Note`) which indicates a lack of low-order reflections. We assume these reflections are probably affected by a beamstop due to a large unit cell of the crystal.

Supplementary Table 1. Crystal data of **3**, **4·18c6**, **5-1**, and **5-2**.

	**3**	**4·18c6**	**5-1**	**5-2**
Solvent	hexane	C_6H_6/hexane	hexane	hexane
Empirical formula	$C_{33}H_{62}Si_4Ge$	$C_{42}H_{75}Ge_3Si_4$ ·$C_{12}H_{24}O_6K$	$C_{66}H_{124}Ge_5Si_8$ ·0.5 hexane	$C_{66}H_{124}Ge_5Si_8$
Formular weight	643.77	1213.56	1548.40	1505.31
Temperature (K)	90(2)	90(2)	90(2)	90(2)
Color	colorless	orange	red	green
Crystal size (mm^3)	0.15×0.15×0.12	0.10×0.10×0.02	0.08x0.05x0.03	0.14×0.12×0.10
Crystal system	monoclinic	monoclinic	monoclinic	triclinic
Space group	$P2_1$ (#4)	$P2_1/c$ (#14)	$P2_1/n$ (#14)	P-1 (#2)
a (Å)	9.1666(6)	21.8091(18)	21.607(4)	13.5498(7)
b (Å)	20.2477(13)	11.9858(10)	12.913(2)	18.1393(9)
c (Å)	20.9974(15)	24.7786(19)	31.518(6)	19.3126(11)
α (deg)	90	90	90	105.770(3)
β (deg)	94.025(2)	100.249(3)	107.735(6)	110.334(3)
γ (deg)	90	90	90	101.994(3)
V (Å^3)	3887.6(5)	6373.8(9)	8376(3)	4036.0(4)
Z	4	4	4	2
D (g/cm^3)	1.100	1.265	1.228	1.239
μ (mm^{-1})	0.930	1.588	1.922	1.993
θ range (deg)	1.95 to 27.50	2.05 to 27.48	1.88 to 27.49	2.03 to 27.49
reflections collected	81114	183458	261942	193427
Independent reflections	17431	14620	19200	18510
R_{int}	0.0471	0.0465	0.0635	0.0803
No. of restrains	1	0	5	0
No. of parameters	753	634	826	778
Completeness to θ (%)	99.3	99.9	99.9	100.0
R_1/wR_2	0.0287/0.0722	0.0238/0.0591	0.0289/0.0704	0.0323/0.0790
Goodness-of-fit on F^2	1.081	1.041	1.060	1.040
Largest diff. peak (e·Å^3)	0.441	0.534	1.310	0.937
Largest diff. hole (e·Å^3)	−0.242	−0.308	−0.422	−0.541
CCDC	2246032	2246033	2268057	2246034

Supplementary Table 2. Crystal data of **6** and **7**.

	**6·NHC**	**7**
Solvent	C_6H_6/hexane	hexane
Empirical formula	$C_{44}H_{82}Ge_2N_2Si_4$	$C_{72}H_{147}Br_3Ge_5Si_{12}$ ·hexane
Formular weight	896.65	2038.82
Temperature (K)	90(2)	90(2)
Color	yellow	red
Crystal size (mm^3)	0.08×0.07×0.02	0.14×0.13×0.08
Crystal system	orthorhombic	triclinic
Space group	$Pna2_1$ (#33)	P-1 (#2)
a (Å)	17.9993(19)	13.5161(7)
b (Å)	28.124(3)	18.6064(11)
c (Å)	9.7643(11)	43.231(2)
α (deg)	90	83.579(2)
β (deg)	90	82.454(2)
γ (deg)	90	77.682(2)
V (Å^3)	4942.8(9)	10490.2(10)
Z	4	4
D (g/cm^3)	1.205	1.291
μ (mm^{-1})	1.343	2.728
θ range (deg)	2.21 to 27.52	1.83 to 27.49
reflections collected	82603	584231
Independent reflections	11354	48123
R_{int}	0.0853	0.0566
No. of restrains	1	0
No. of parameters	494	1957
Completeness to θ (%)	99.9	99.9
R_1/wR_2	0.0294/0.0679	0.0304/0.0701
Goodness-of-fit on F^2	1.058	1.106
Largest diff. peak (e·Å^3)	0.6018	1.152
Largest diff. hole (e·Å^3)	−0.312	−0.594
CCDC	2246035	2246036

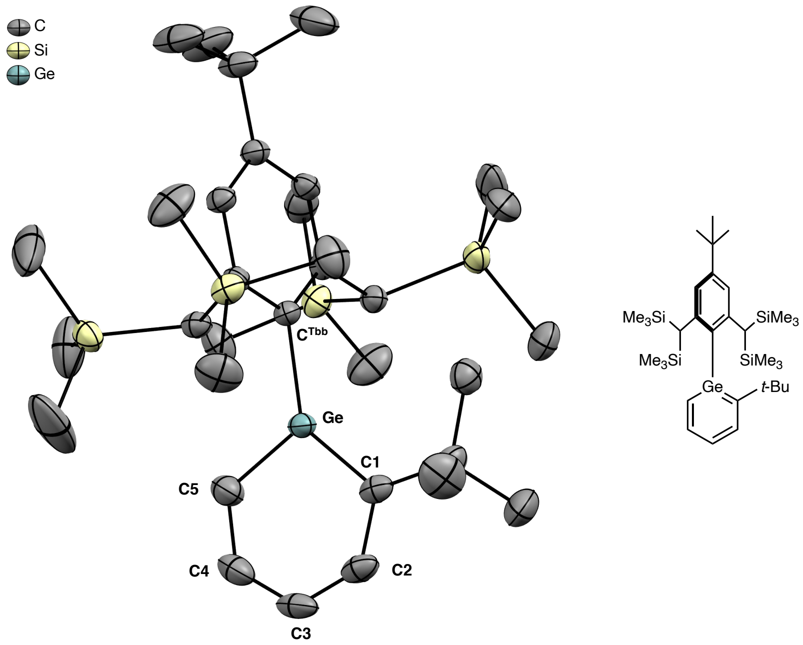

Supplementary Fig. 1 Atomic displacement parameter plot for **3-Ge** at 50% probability.

Supplementary Table 3. Selected structural parameters of **3-Ge**.

	Distance (Å)		Angle (°)
Ge–C1	1.859(3)	C5–Ge–C1	106.3(1)
C1–C2	1.399(5)	Ge–C1–C2	114.2(2)
C2–C3	1.388(5)	C1–C2–C3	129.2(3)
C3–C4	1.384(5)	C2–C3–C4	125.9(3)
C4–C5	1.377(5)	C3–C4–C5	125.3(3)
C5–Ge	1.842(3)	C4–C5–Ge1	118.9(2)
Ge–C^{Tbb}	1.946(3)		

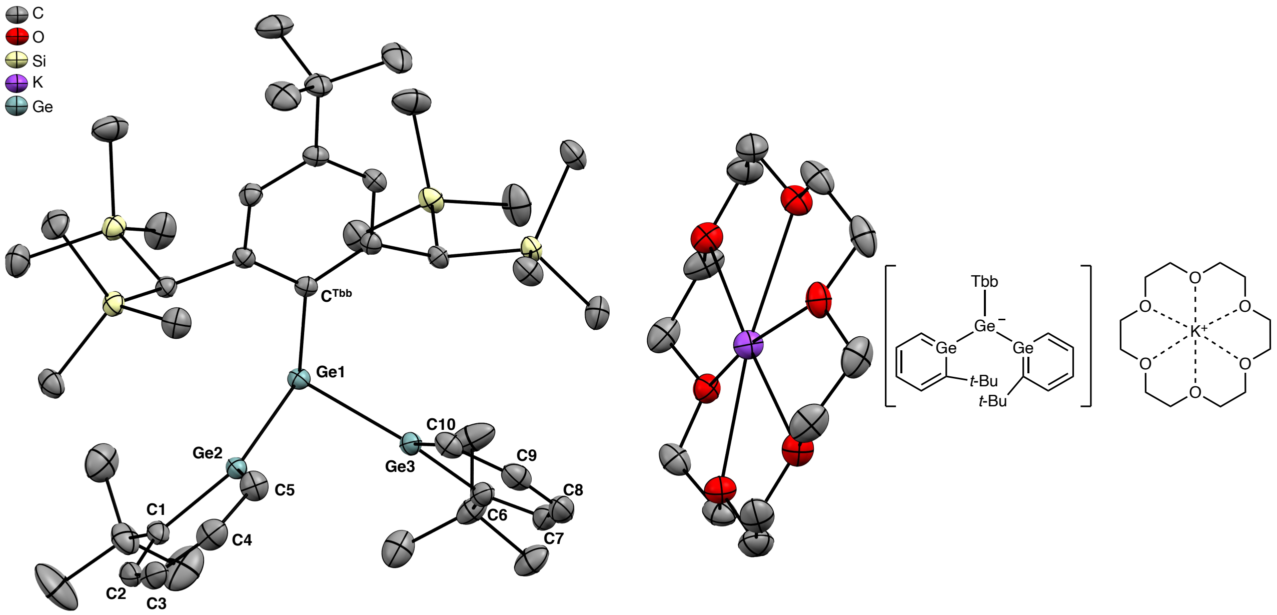

Supplementary Fig. 2. Atomic displacement parameter plot for **4·18c6** at 50% probability.

Supplementary Table 4. Selected strucural parameters of **4·18c6**.

	Distance (Å)		Angle (°)
Ge1–Ge2	2.5078(5)	C5–Ge2–C1	102.33(7)
Ge1–Ge3	2.4913(6)	Ge2–C1–C2	117.4(1)
Ge1–C^{Tbb}	2.063(1)	C1–C2–C3	128.4(1)
Ge2–C1	1.892(2)	C2–C3–C4	125.4(2)
C1–C2	1.388(2)	C3–C4–C5	124.6(2)
C2–C3	1.399(2)	C4–C5–Ge2	121.8(1)
C3–C4	1.389(3)	C10–Ge3–C6	102.78(7)
C4–C5	1.383(2)	Ge3–C6–C7	117.4(1)
C5–Ge2	1.857(1)	C6–C7–C8	128.2(2)
Ge3–C6	1.887(1)	C7–C8–C9	125.2(2)
C6–C7	1.388(2)	C8–C9–C10	125.1(2)
C7–C8	1.403(2)	C9–C10–Ge3	121.2(1)
C8–C9	1.386(2)		
C9–C10	1.386(2)		
C10–Ge3	1.854(2)		
Ge1–K	8.3923(8)		

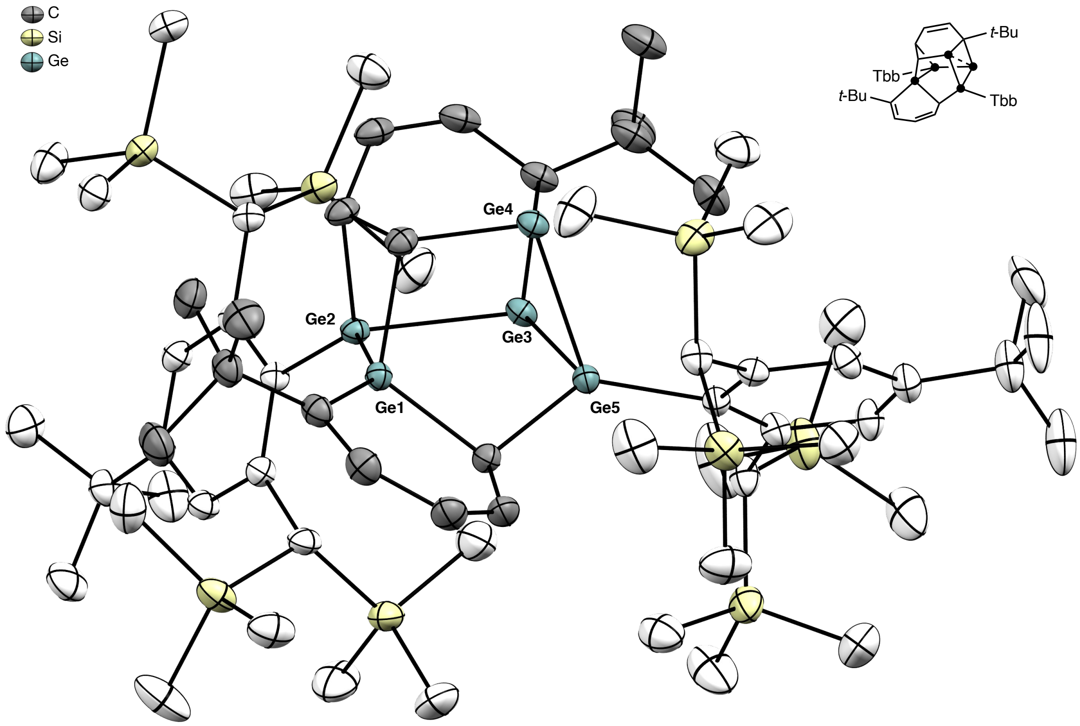

Supplementary Fig. 3. Atomic displacement parameter plot for **5-1** at 50% probability.

Supplementary Table 5. Ge–Ge distances of **5-1**.

	Distance (Å)
Ge1–Ge2	2.4486(5)
Ge2–Ge3	2.518(1)
Ge3–Ge4	2.7849(8)
Ge4–Ge5	2.5156(5)
Ge3–Ge5	2.4391(6)

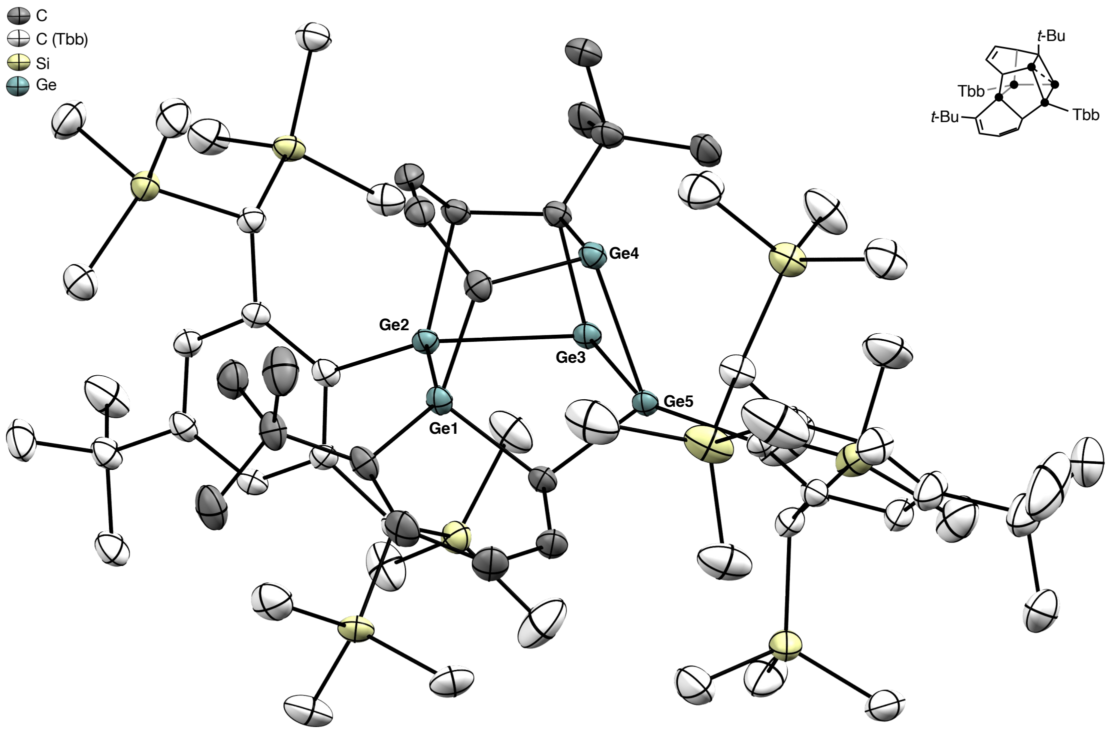

Supplementary Fig. 4. Atomic displacement parameter plot for **5-2** at 50% probability.

Supplementary Table 6. Ge–Ge distances of **5-2**.

	Distance (Å)
Ge1–Ge2	2.4414(4)
Ge2–Ge3	2.5061(6)
Ge3–Ge4	2.8648(5)
Ge4–Ge5	2.5072(4)
Ge3–Ge5	2.4450(4)

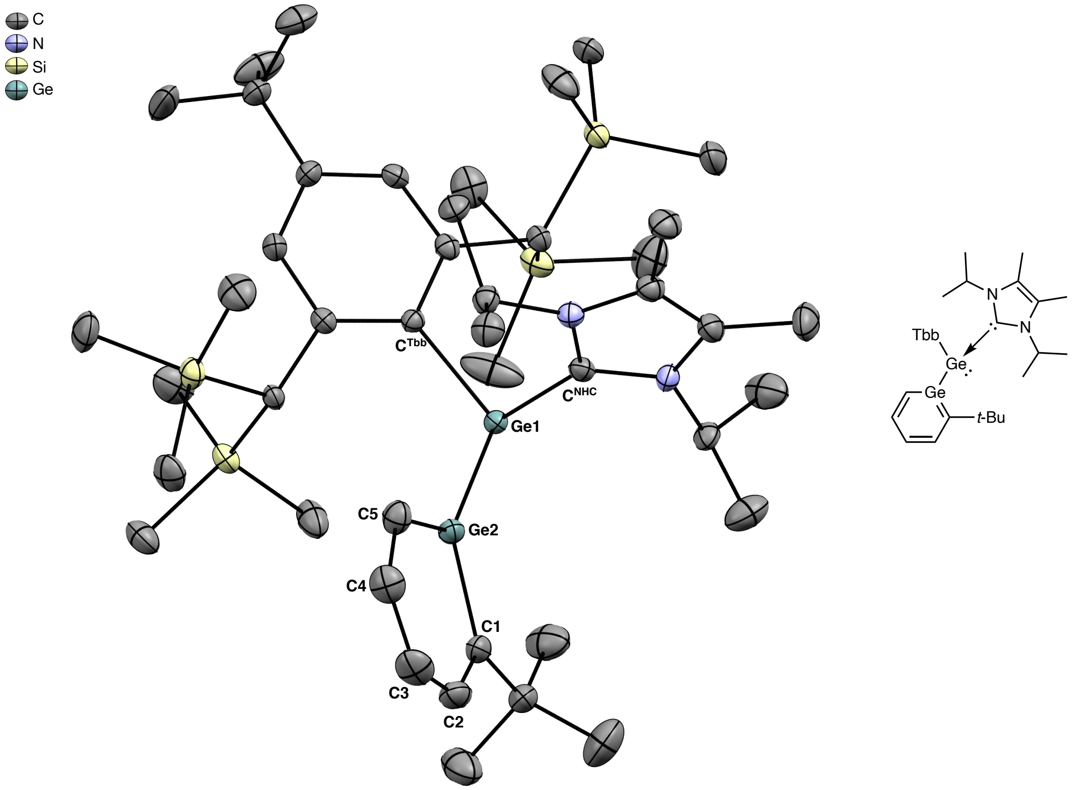

Supplementary Fig. 5. Atomic displacement parameter plot for **6·NHC** at 50% probability.

Supplementary Table 7. Selected structural parameters of **6·NHC**.

	Distance (Å)		Angle (°)
Ge1–Ge2	2.5257(8)	Ge2–Ge1–C^{NHC}	89.69(8)
Ge1–C^{Tbb}	2.049(3)	Ge2–Ge1–C^{Tbb}	113.08(8)
Ge1–C^{NHC}	2.095(3)	C5–Ge2–C1	102.5(1)
Ge2–C1	1.888(3)	Ge2–C1–C2	116.4(2)
C1–C2	1.392(5)	C1–C2–C3	129.6(3)
C2–C3	1.388(6)	C2–C3–C4	125.2(4)
C3–C4	1.391(6)	C3–C4–C5	124.5(4)
C4–C5	1.376(6)	C4–C5–Ge2	121.8(3)
C5–Ge2	1.870(4)		

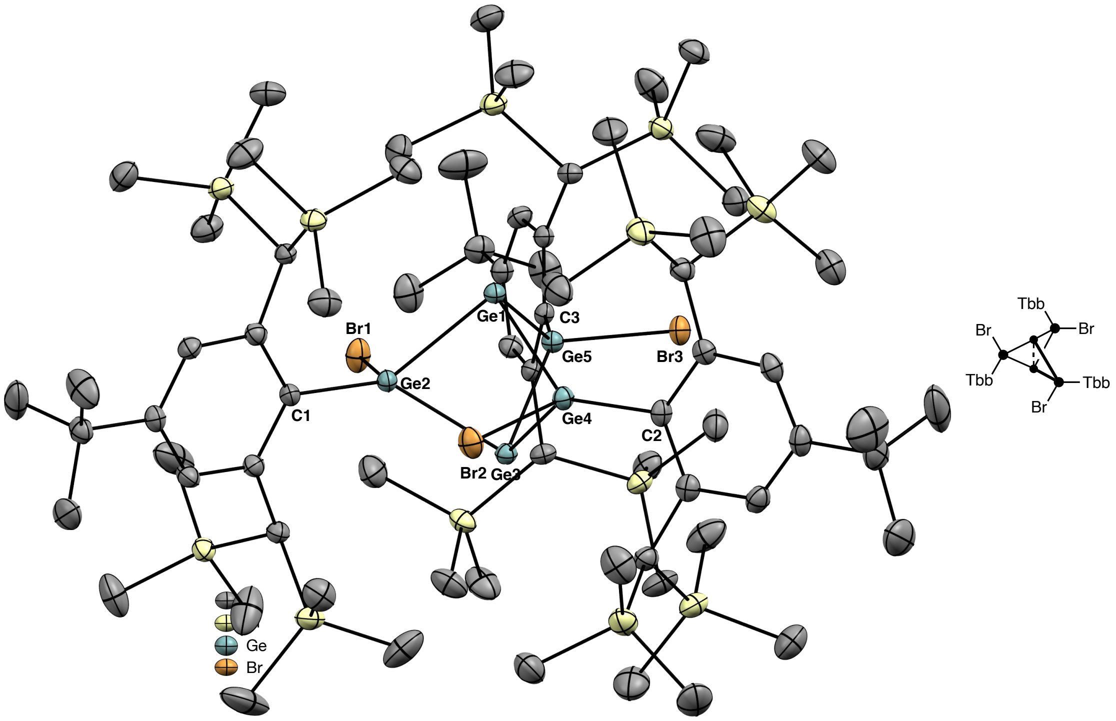

Supplementary Fig. 6. Atomic displacement parameter plot for **7** at 50% probability.

Supplementary Table 8. Selected structural parameters of **7**.

	Distance (Å)		Angle (°)
Ge1–Ge2	2.4698(7)	Ge3–Ge2–Ge1	73.17(2)
Ge1–Ge4	2.499(1)	Ge3–Ge4–Ge1	73.17(2)
Ge1–Ge5	2.4950(8)	Ge3–Ge5–Ge1	73.29(2)
Ge3–Ge2	2.5123(7)		
Ge3–Ge4	2.476(1)		
Ge3–Ge5	2.4875(8)		
Ge1–Ge3	2.9697(8)		
Ge2–C1	1.963(2)		
Ge4–C2	1.955(2)		
Ge5–C3	1.963(2)		
Ge2–Br1	2.3599(8)		
Ge4–Br2	2.364(1)		
Ge5–Br3	2.370(1)		

4. NMR spectra of new compounds

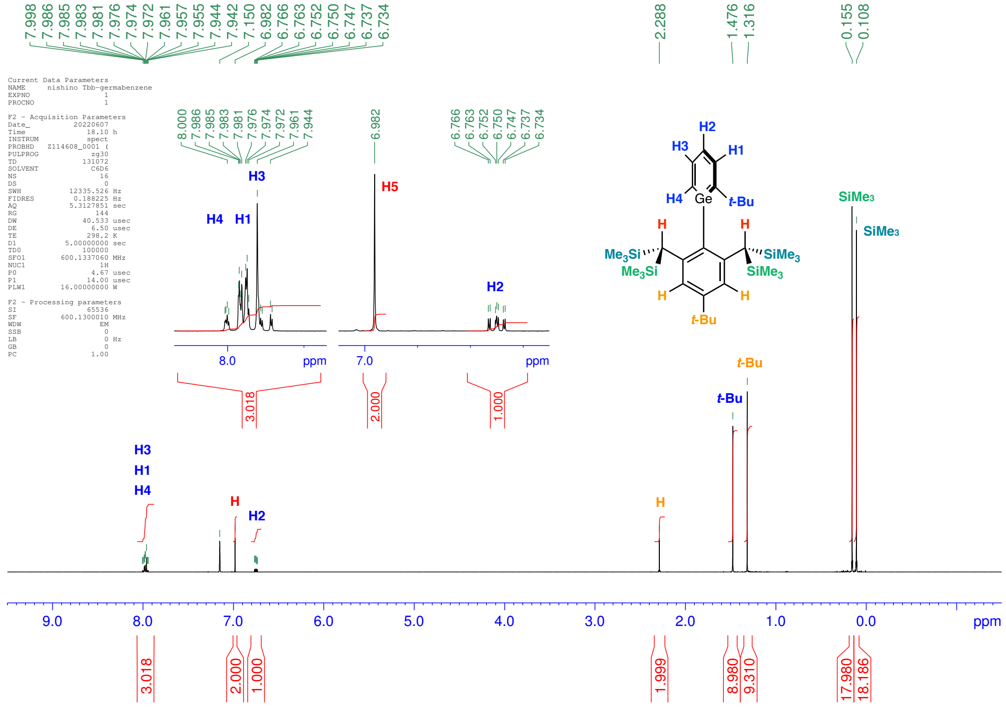

Supplementary Fig. 7. ^{1}H NMR spectrum of **3-Ge** (C_6D_6, 600 MHz, r.t.).

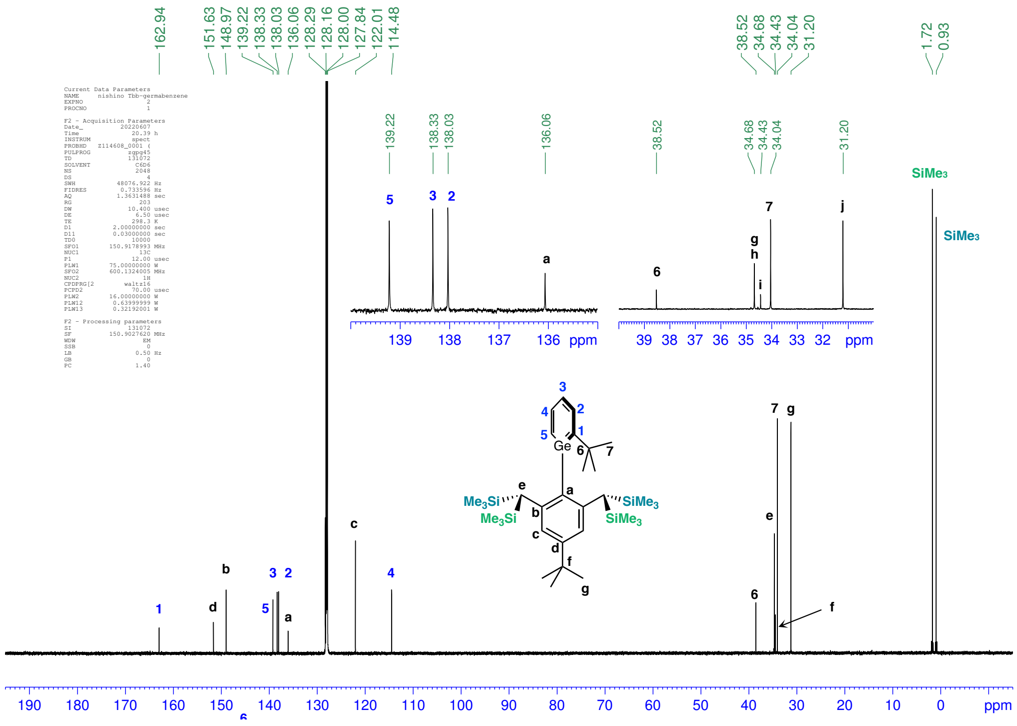

Supplementary Fig. 8. $^{13}C\{^{1}H\}$ NMR spectrum of **3-Ge** (C_6D_6, 150 MHz, r.t.).

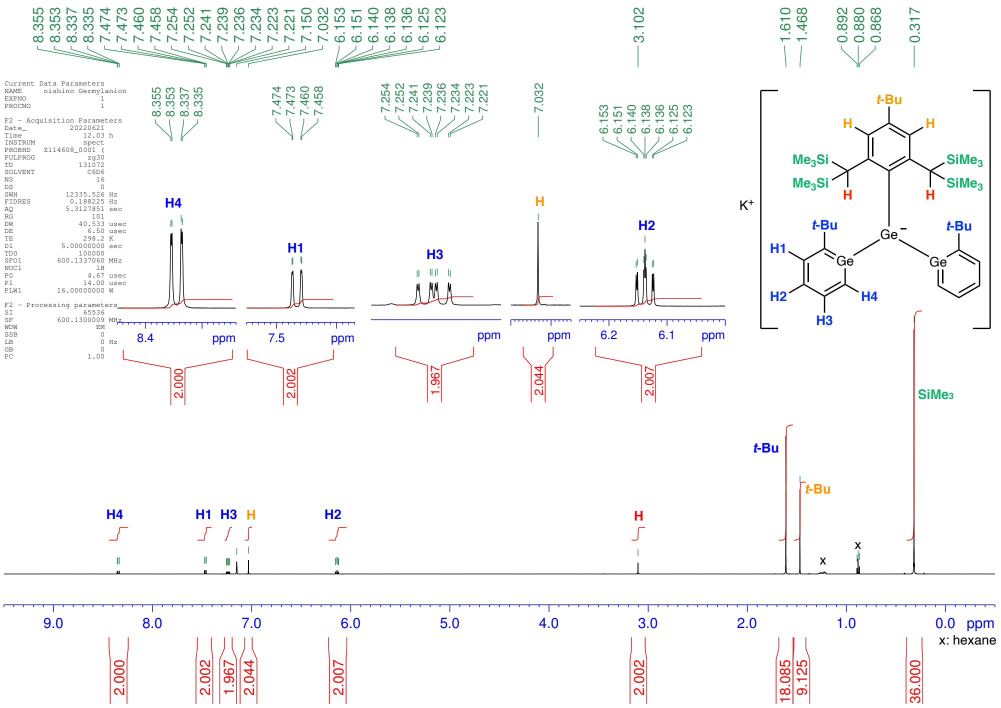

Supplementary Fig. 9. ^{1}H NMR spectrum of **4** (C_6D_6, 600 MHz, r.t.).

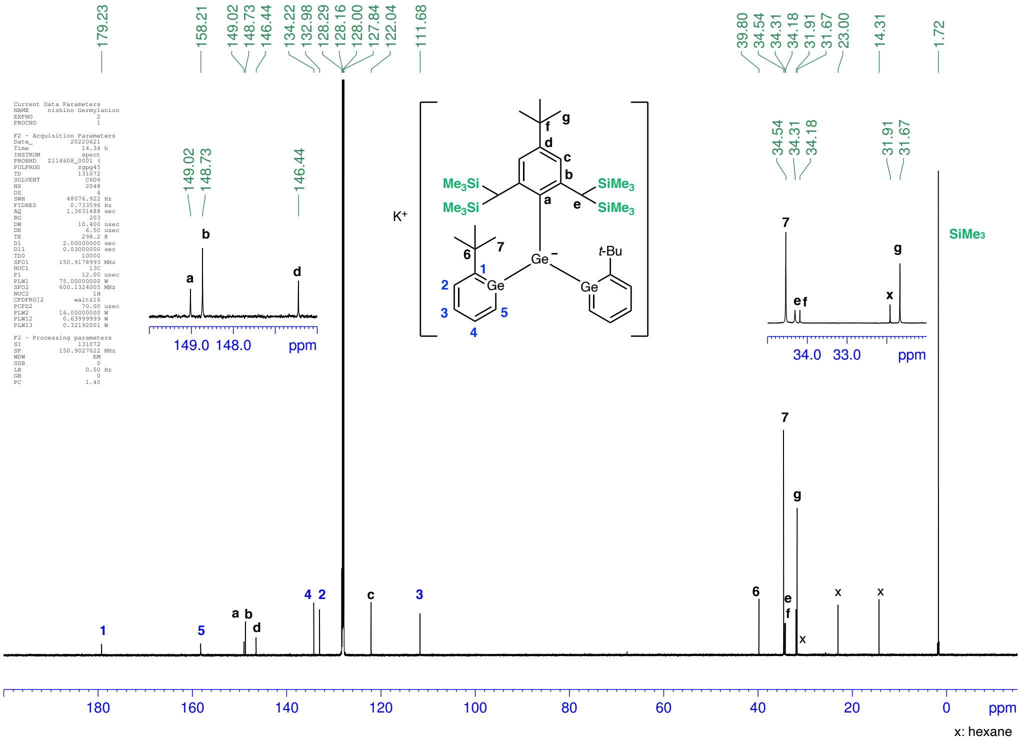

Supplementary Fig. 10. ^{13}C{^{1}H} NMR spectrum of **4** (C_6D_6, 150 MHz, r.t.).

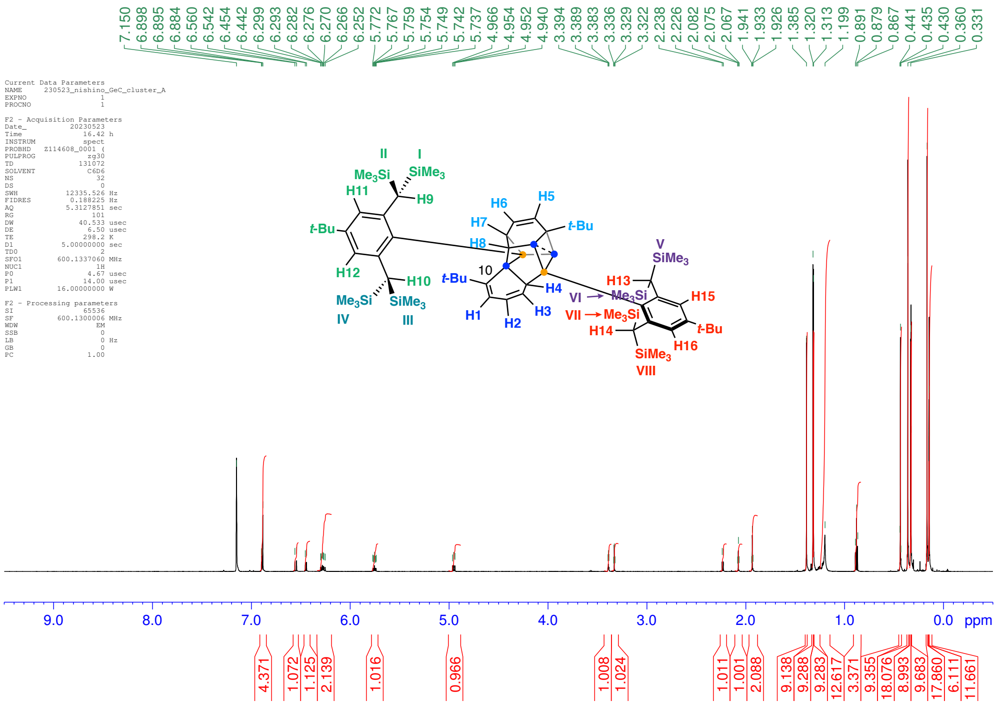

Supplementary Fig. 11. ^{1}H NMR spectrum of **5-1** (C_6D_6, 600 MHz, r.t.).

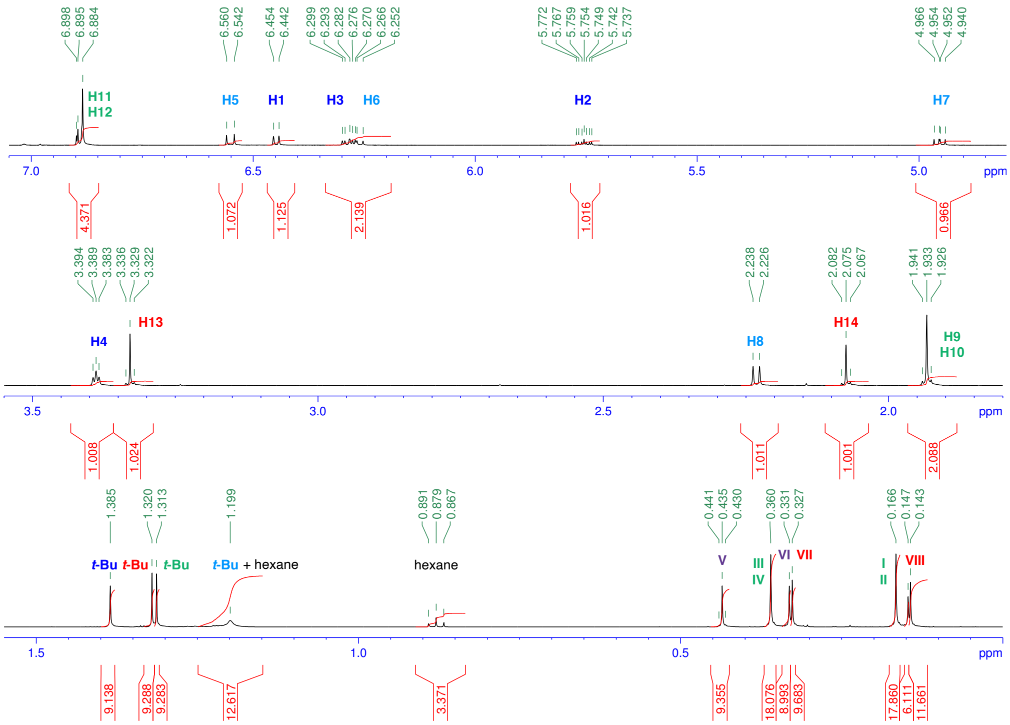

Supplementary Fig. 12. Magnified ^{1}H NMR spectra of **5-1** on 0–1.6, 1.6–3.6, and 4.6–7.1 ppm (C_6D_6, 600 MHz, r.t.).

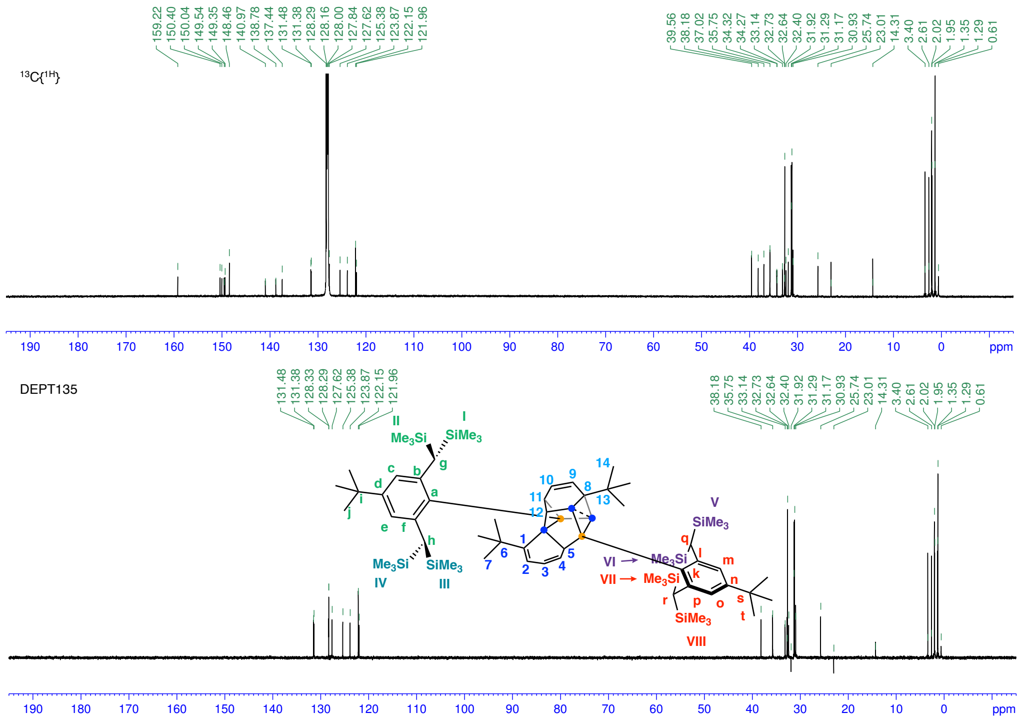

Supplementary Fig. 13. $^{13}C\{^1H\}$ and ^{13}C DEPT135 NMR spectrum of **5-1** (C_6D_6, 150 MHz, r.t.).

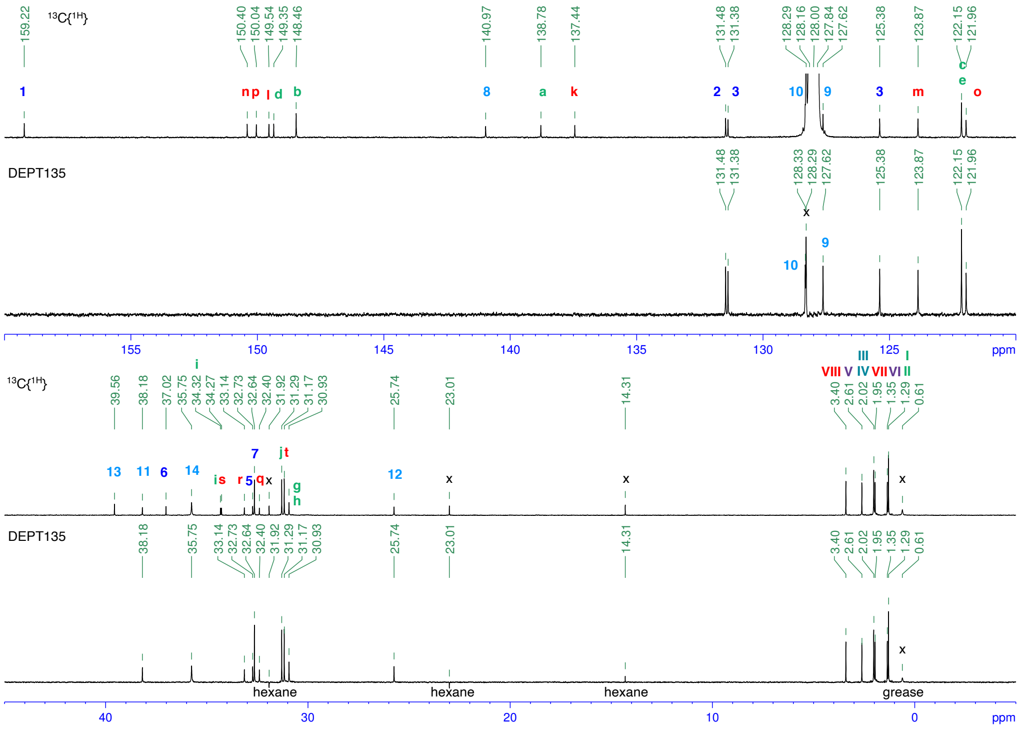

Supplementary Fig. 14. Magnified $^{13}C\{^1H\}$ and ^{13}C DEPT135 NMR spectra of **5-1** on 0–50 and 120–160 ppm (C_6D_6, 150 MHz, r.t.).

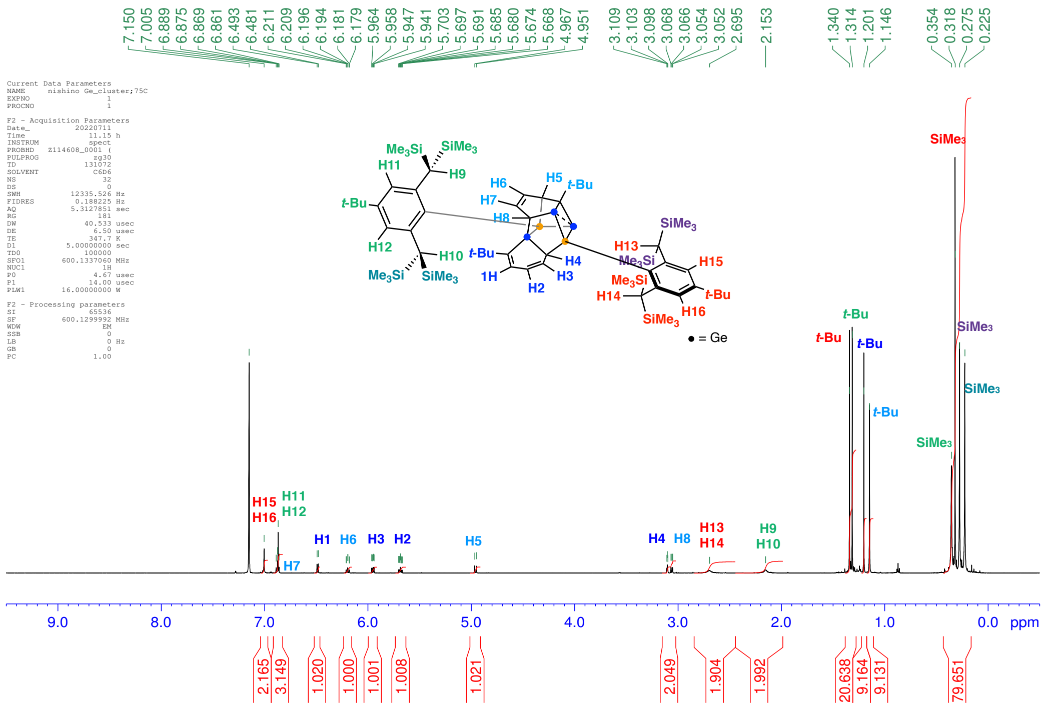

Supplementary Fig. 15. ^{1}H NMR spectrum of **5-2** (C_6D_6, 600 MHz, 348 K).

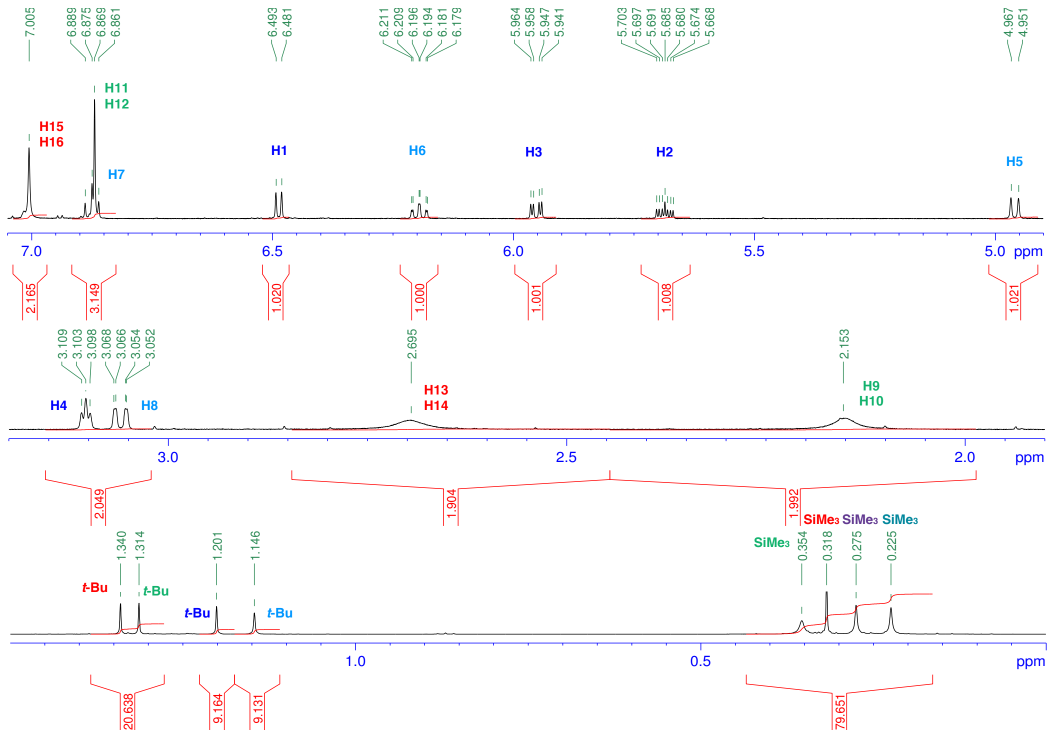

Supplementary Fig. 16. Magnified ^{1}H NMR spectra of **5-2** on 0–1.5, 1.8–3.5, and 4.8–7.1 ppm (C_6D_6, 600 MHz, 348 K).

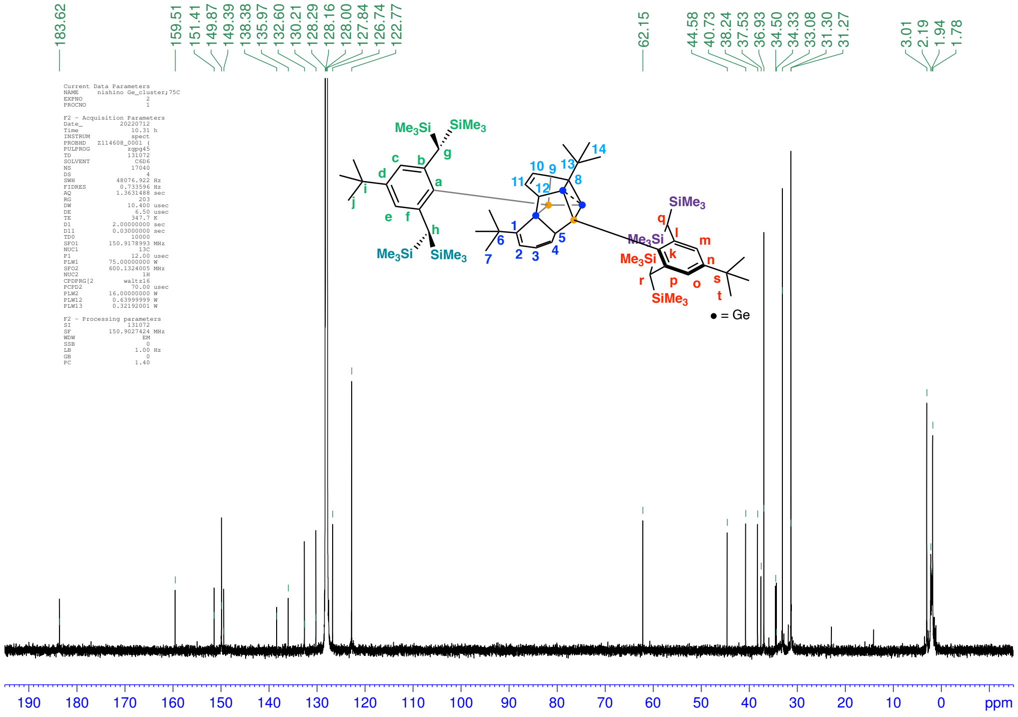

Supplementary Fig. 17. $^{13}C\{^{1}H\}$ NMR spectrum of **5-2** (C_6D_6, 150 MHz, 348 K).

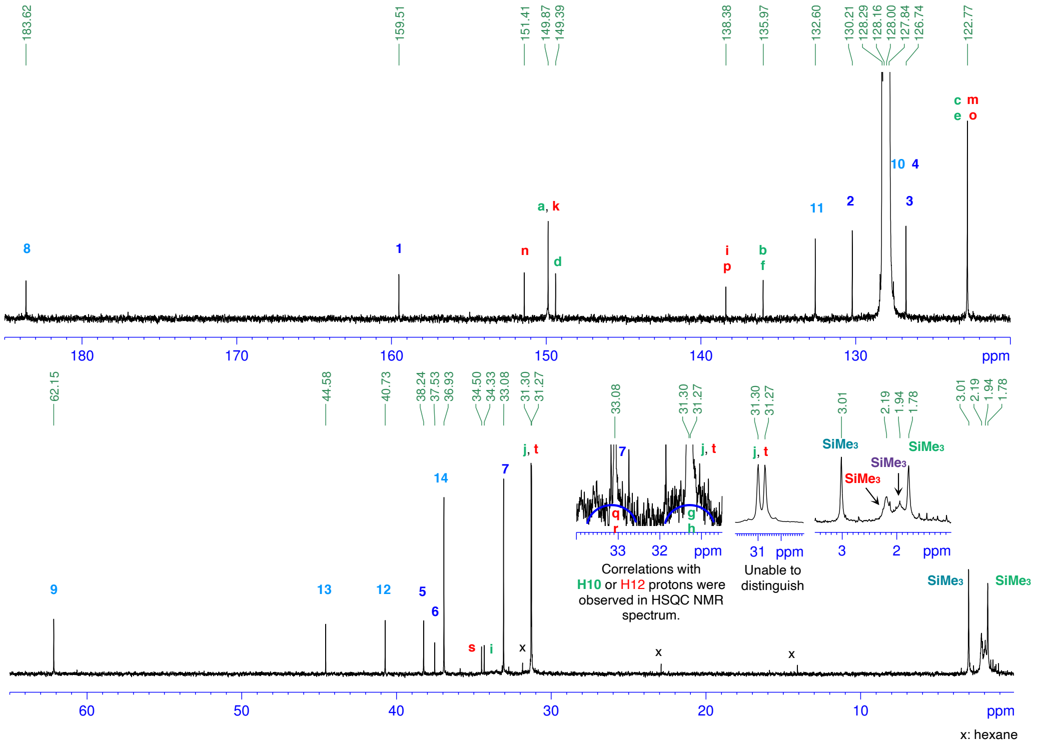

Supplementary Fig. 18. Magnified $^{13}C\{^{1}H\}$ NMR spectra of **5-2** on 0–60 and 120–185 ppm (C_6D_6, 150 MHz, 348 K).

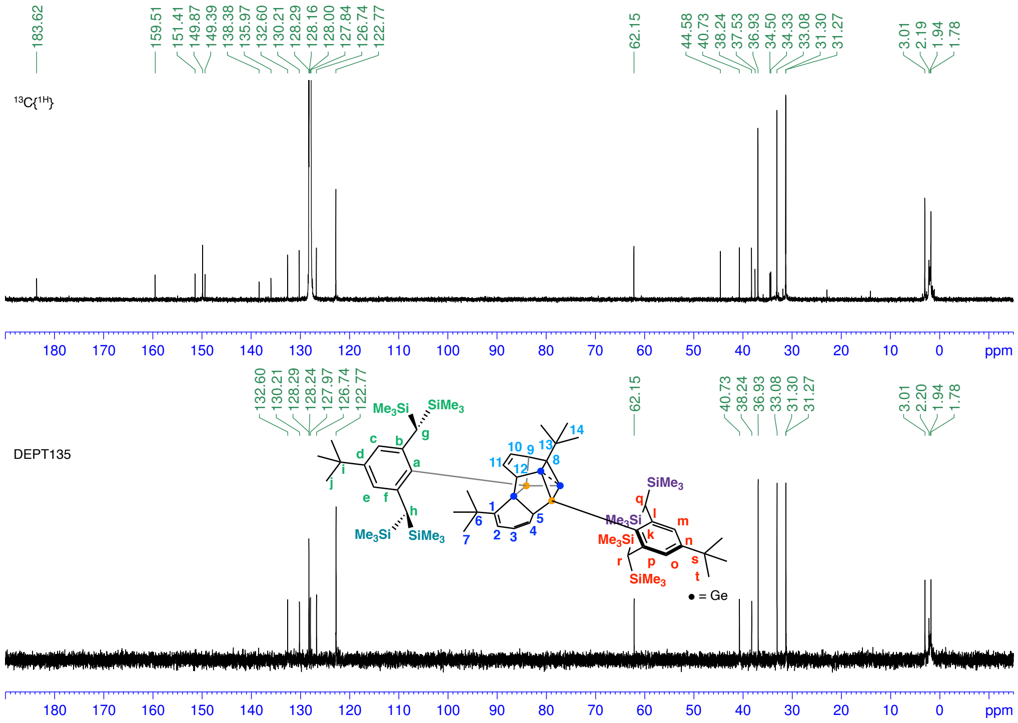

Supplementary Fig. 19. ^{13}C DEPT135 NMR spectrum of **5-2** (C_6D_6, 150 MHz, 348 K).

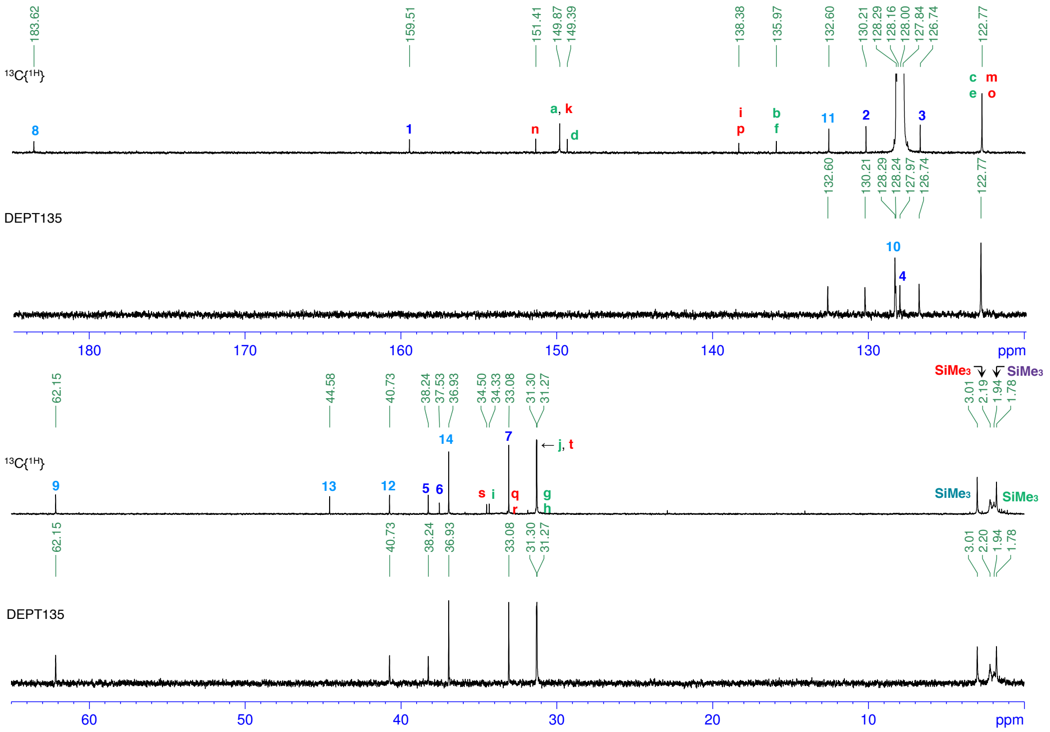

Supplementary Fig. 20. Magnified ^{13}C DEPT135 NMR spectra of **5-2** on 0–60 and 120–185 ppm (C_6D_6, 150 MHz, 348 K).

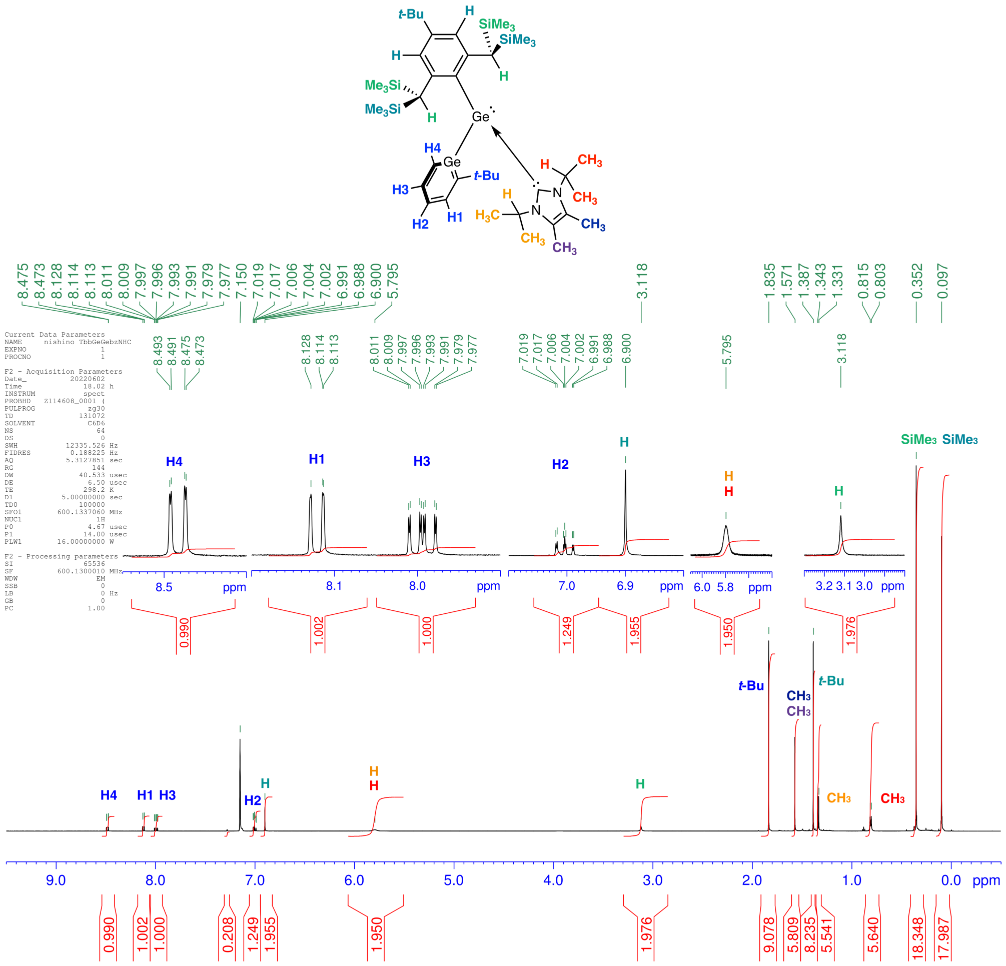

Supplementary Fig. 21. ^{1}H NMR spectrum of **6·NHC** (C_6D_6, 600 MHz, r.t.).

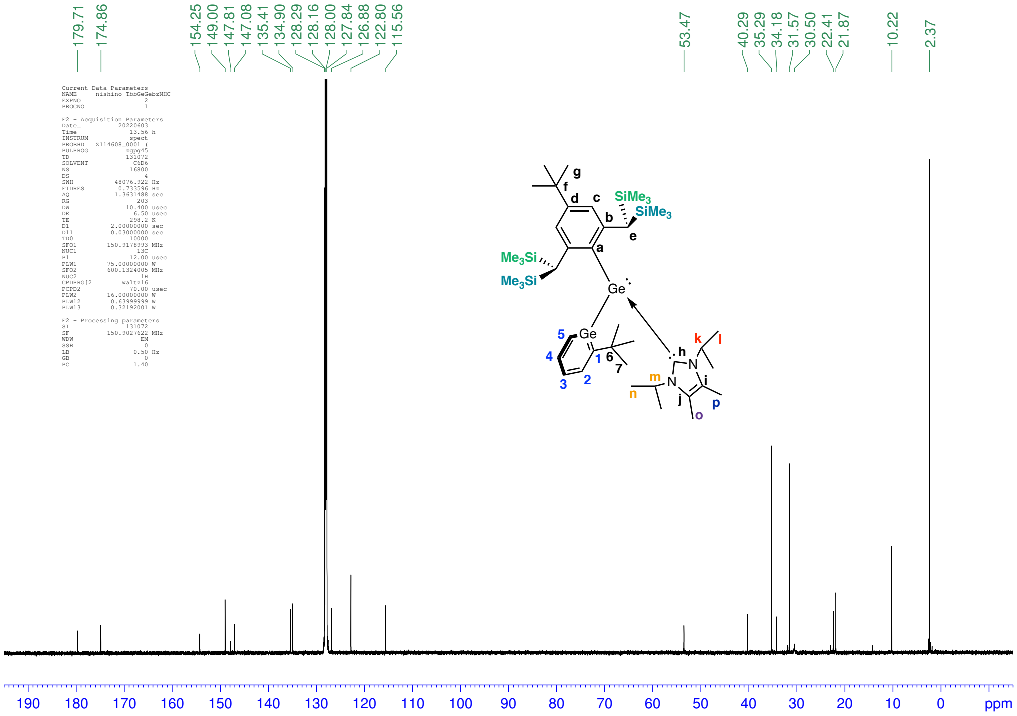

Supplementary Fig. 22. $^{13}C\{^{1}H\}$ NMR spectrum of **6·NHC** (C_6D_6, 150 MHz, r.t.).

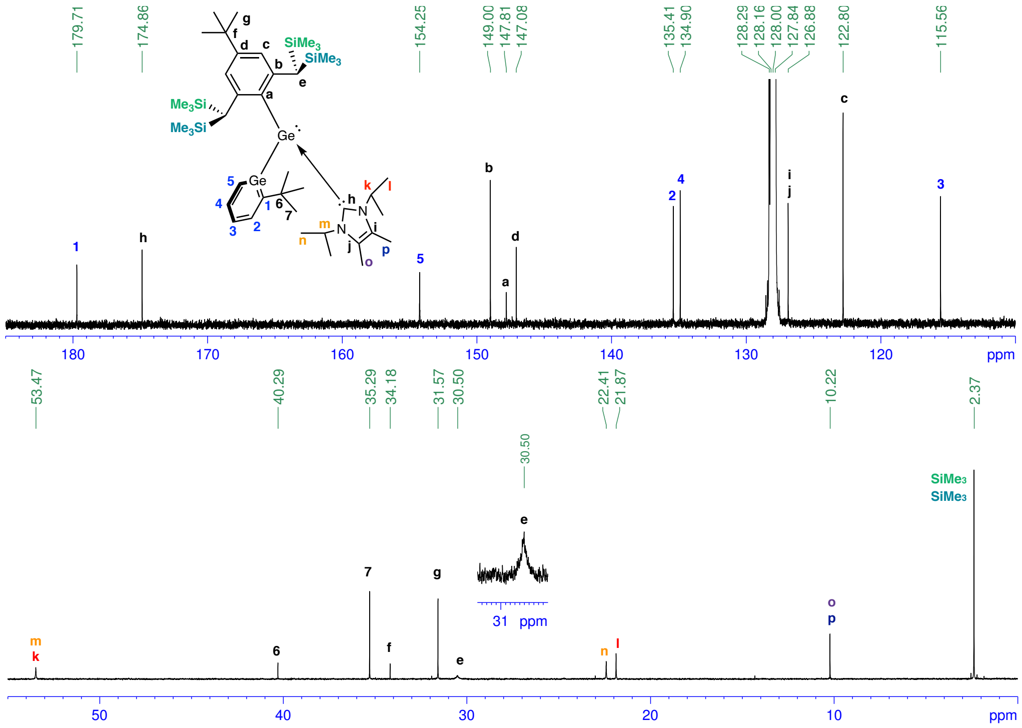

Supplementary Fig. 23. Magnified $^{13}C\{^{1}H\}$ NMR spectra of **6·NHC** on 0–55 and 110–185 ppm (C_6D_6, 150 MHz, r.t.).

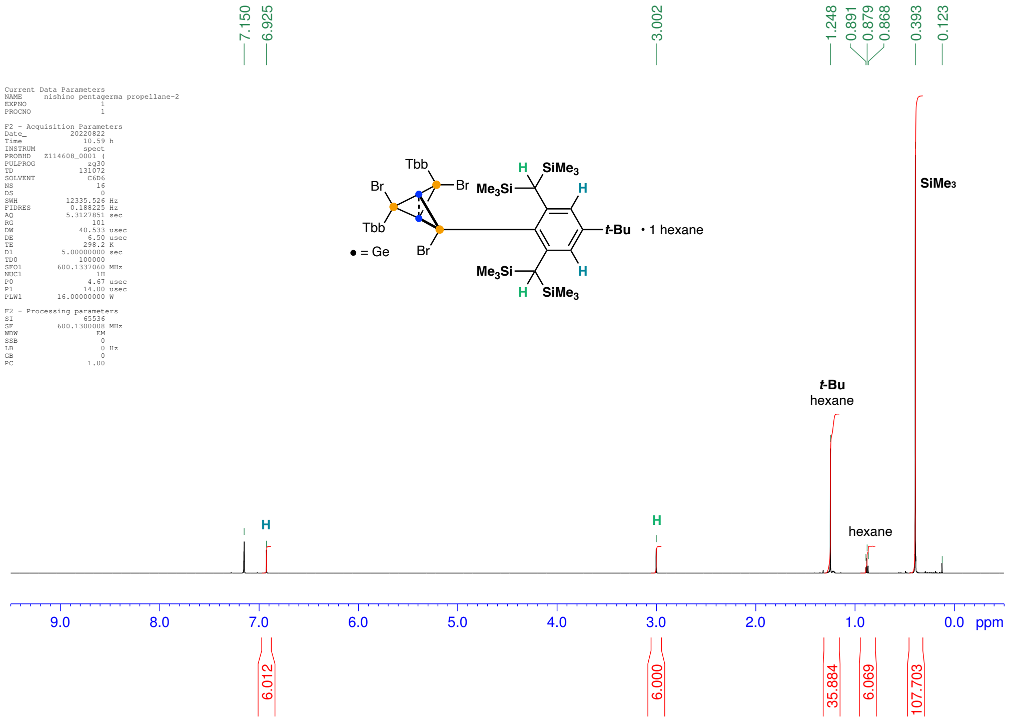

Supplementary Fig. 24. ^{1}H NMR spectrum of **7** (C_6D_6, 600 MHz, r.t.).

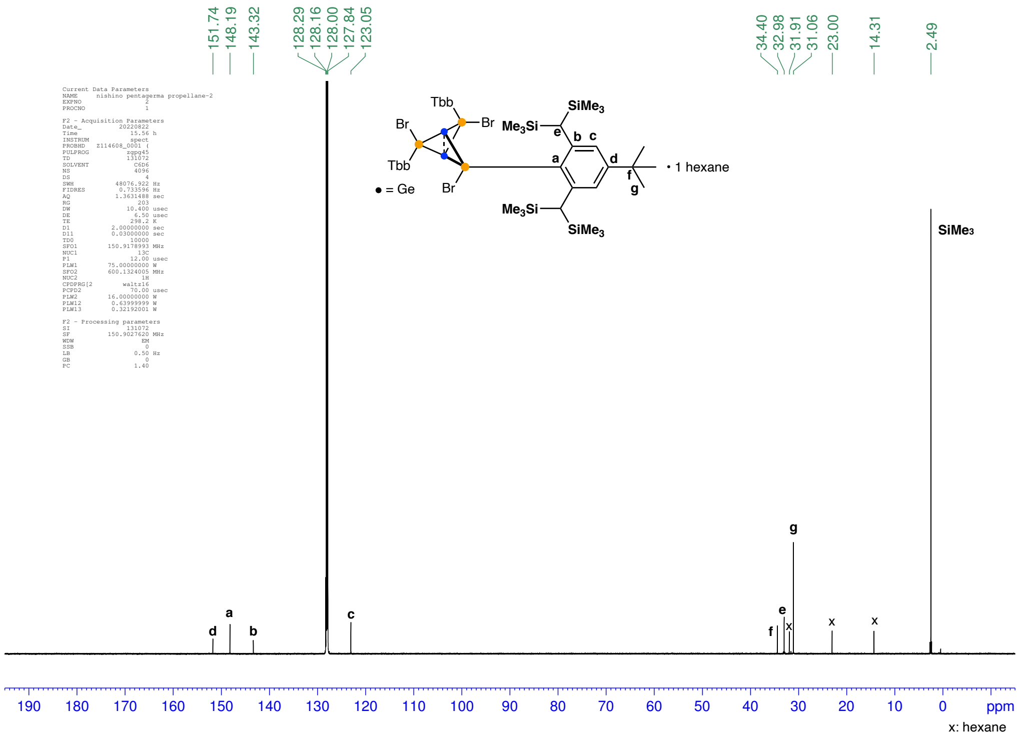

Supplementary Fig. 25. $^{13}C\{^{1}H\}$ NMR spectrum of **7** (C_6D_6, 150 MHz, r.t.).

5. NMR spectra of the reaction products in the mechanistic investigation

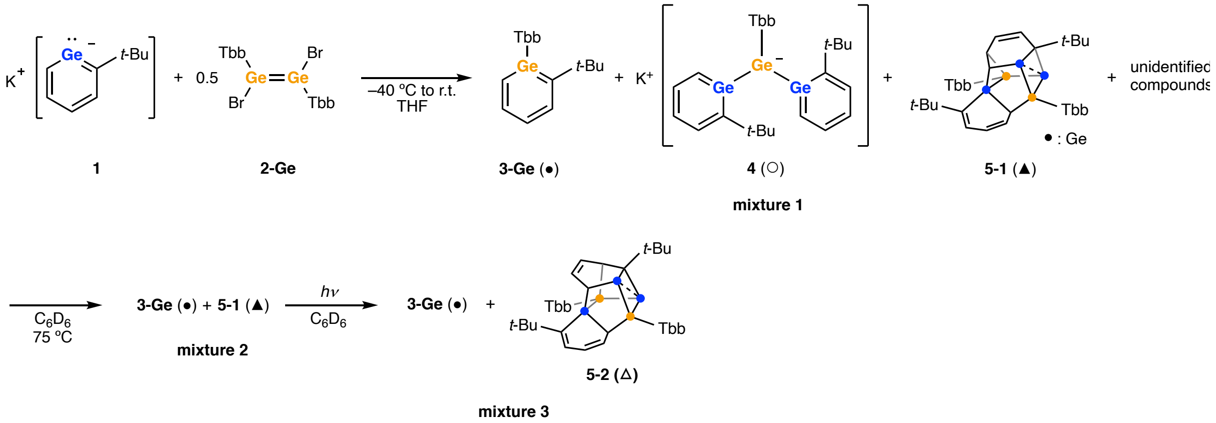

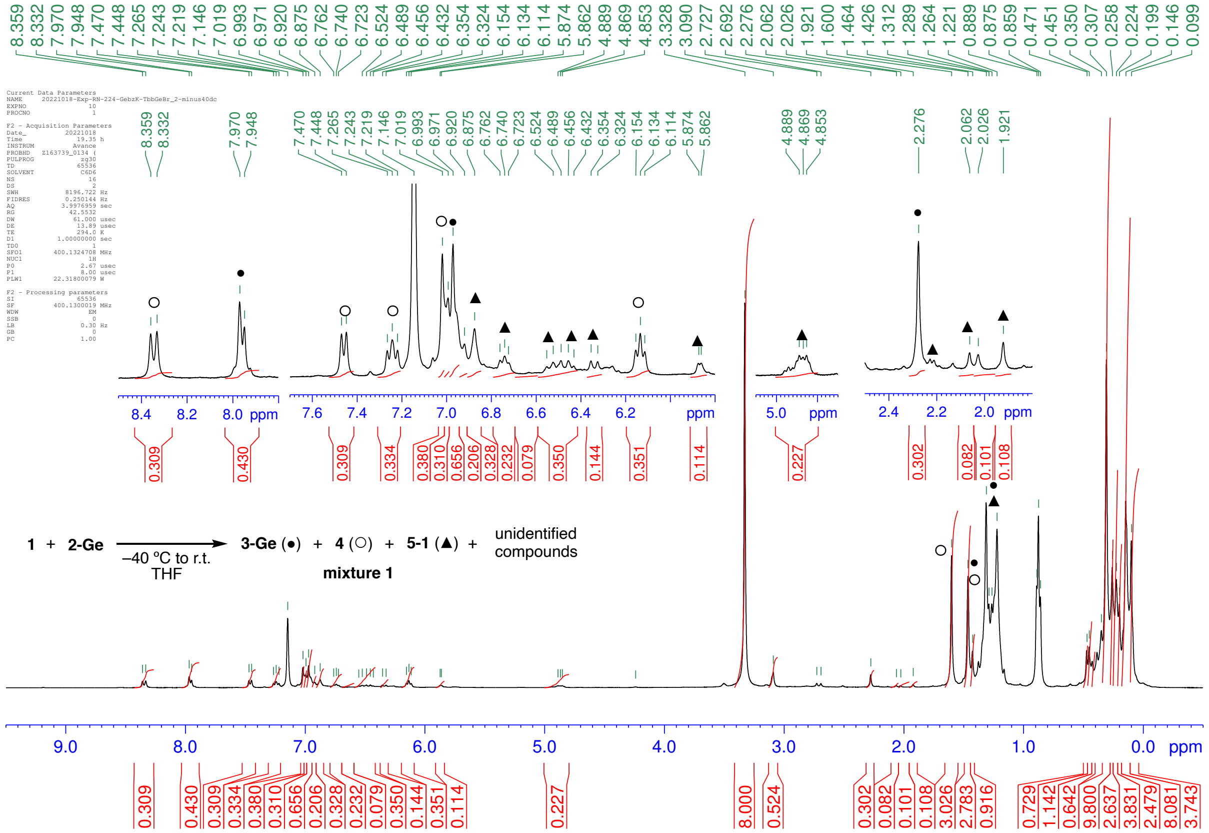

Supplementary Fig. 26. ^{1}H NMR spectrum of **mixture 1** in the reaction of **1** with 0.5 eq. of **2-Ge** (C_6D_6, 400 MHz, r.t.).

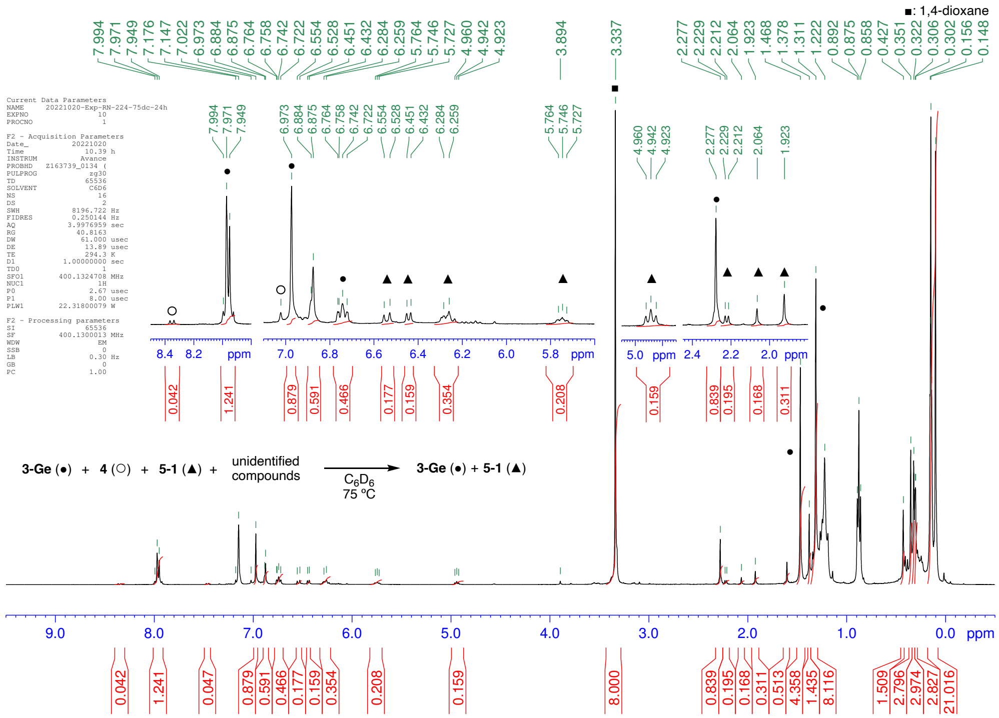

Supplementary Fig. 27. ^{1}H NMR spectrum of **mixture 2** in the reaction of **1** with 0.5 eq. of **2-Ge** (C_6D_6, 400 MHz, r.t.).

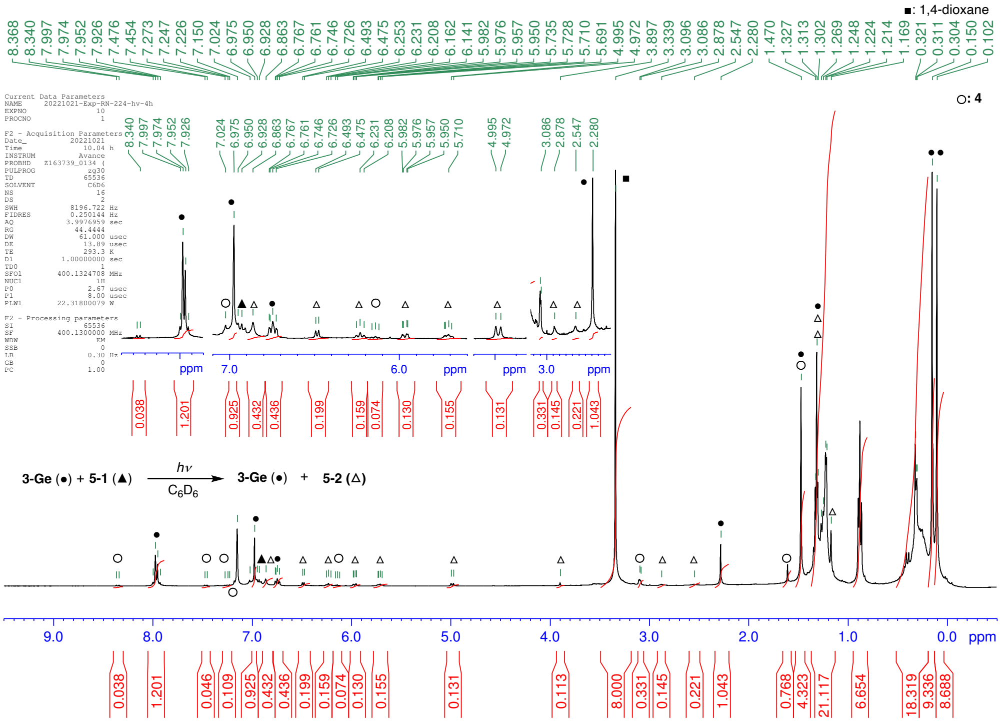

Supplementary Fig. 28. ^{1}H NMR spectrum of **mixture 3** in the reaction of **1** with 0.5 eq. of **2-Ge** (C_6D_6, 400 MHz, r.t.).

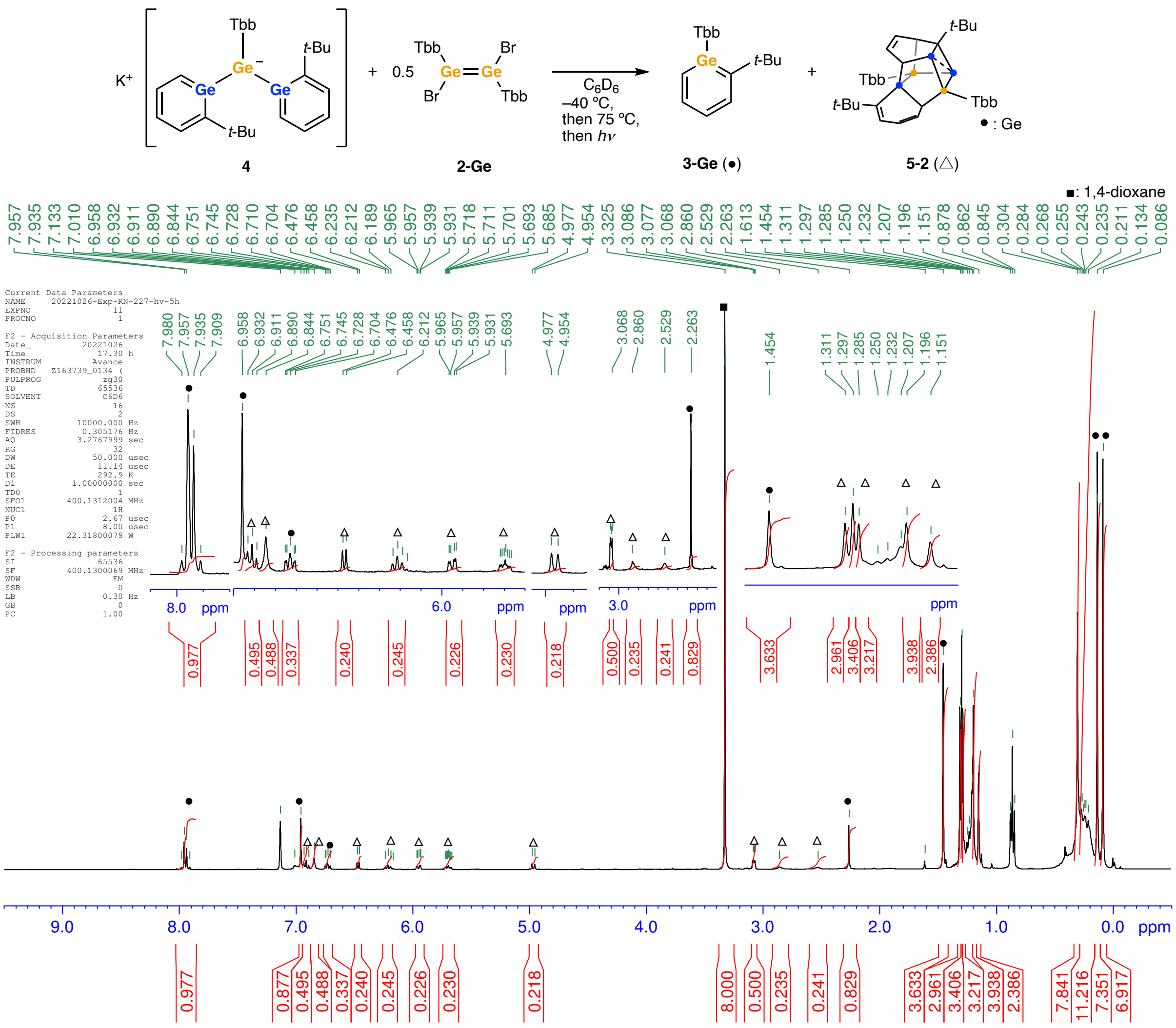

Supplementary Fig. 29. ^{1}H NMR spectrum of the mixture after the reaction of **4** with 0.5 eq. of **2-Ge** (C_6D_6, 400 MHz, r.t.).

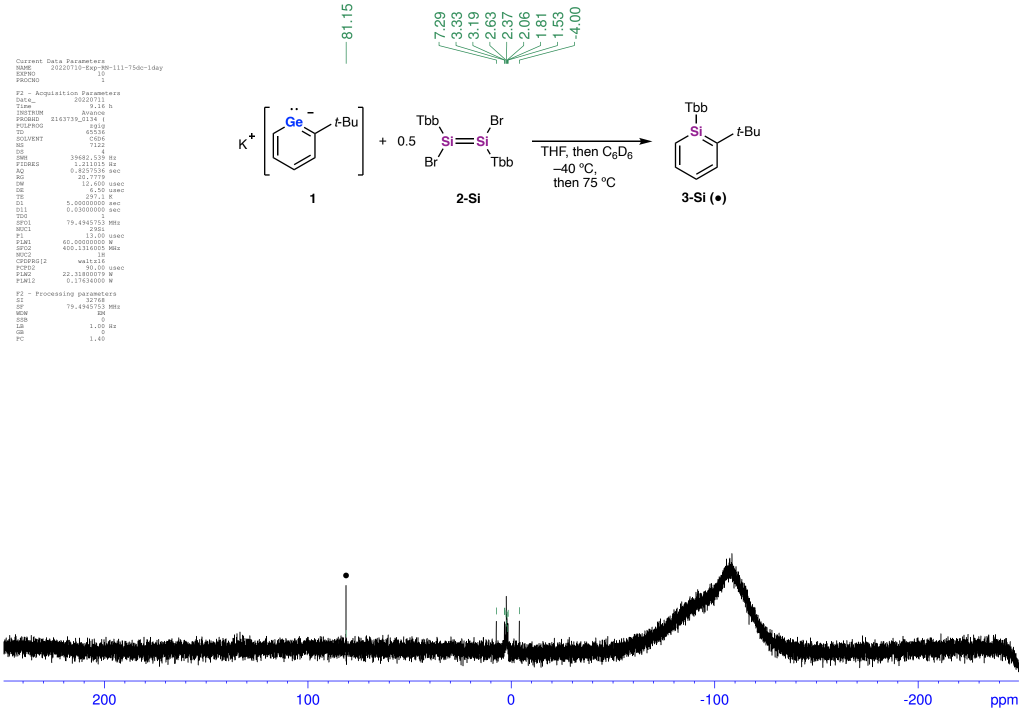

Supplementary Fig. 30. ^{29}Si{^{1}H} NMR spectrum of the mixture after the reaction of **1** with 0.5 eq. of **2-Si** (C_6D_6, 79 MHz, r.t.).

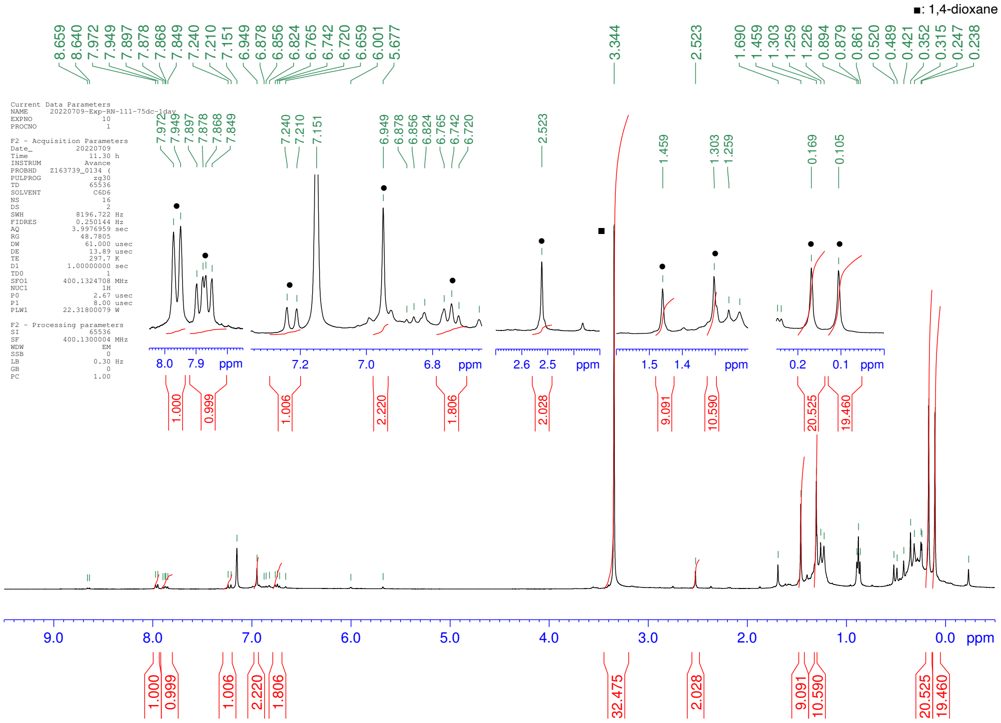

Supplementary Fig. 31. ^{1}H NMR spectrum of the mixture after the reaction of **1** with 0.5 eq. of **2-Si** (C_6D_6, 400 MHz, r.t.).

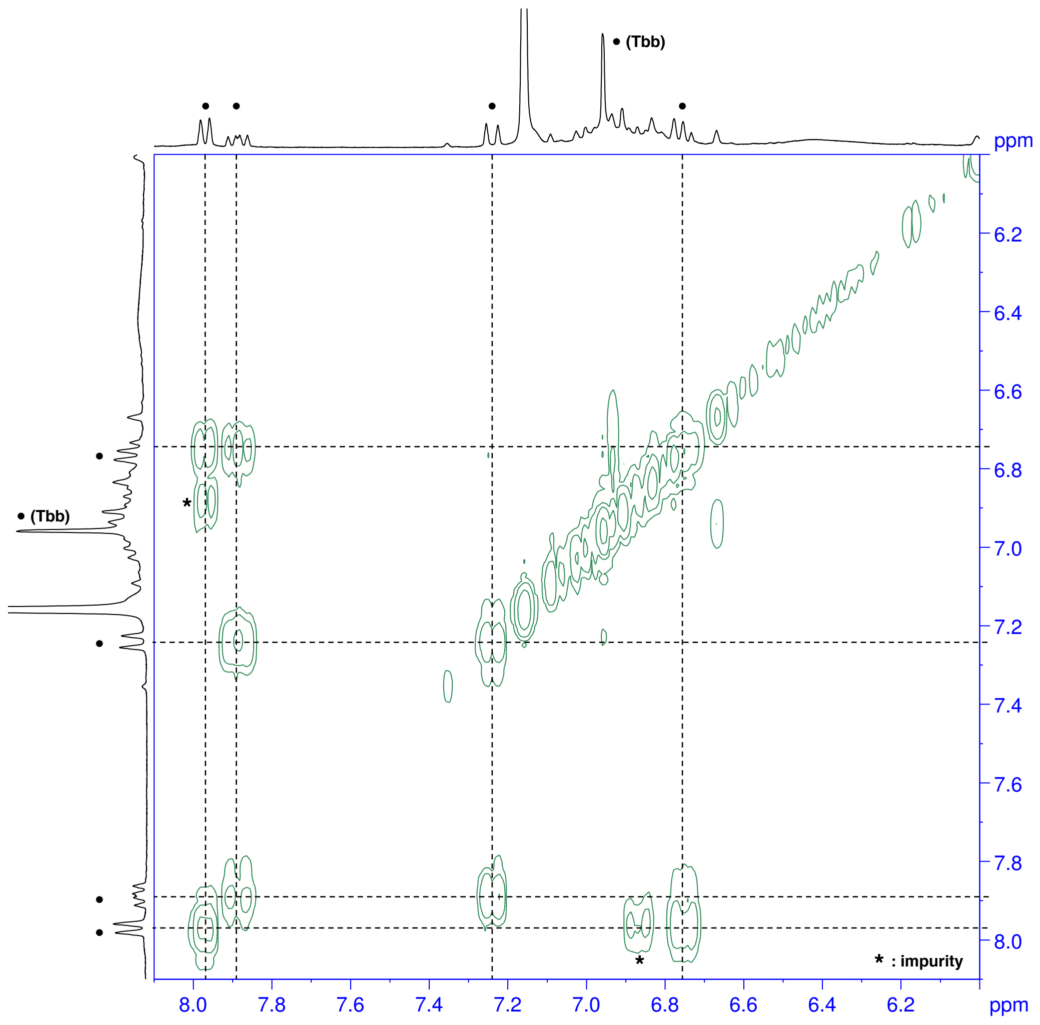

Supplementary Fig. 32. ^{1}H–^{1}H COSY spectrum of the mixture after the reaction of **1** with 0.5 eq. of **2-Si** on 6.0–8.1 ppm (C_6D_6, 400 MHz, r.t.).

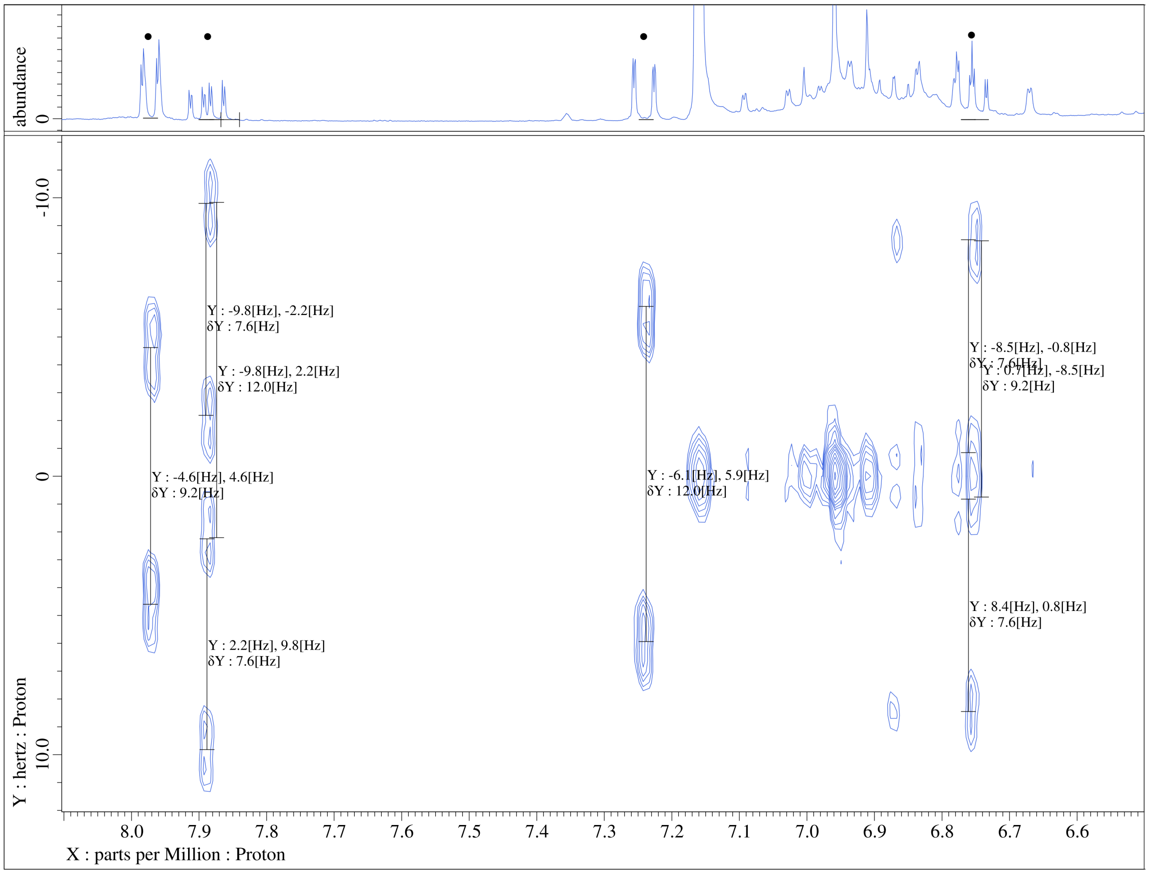

Supplementary Fig. 33. J-resolved spectrum of the mixture after the reaction of **1** with 0.5 eq. of **2-Si** on 6.5–8.1 ppm (C_6D_6, 400 MHz, r.t.).

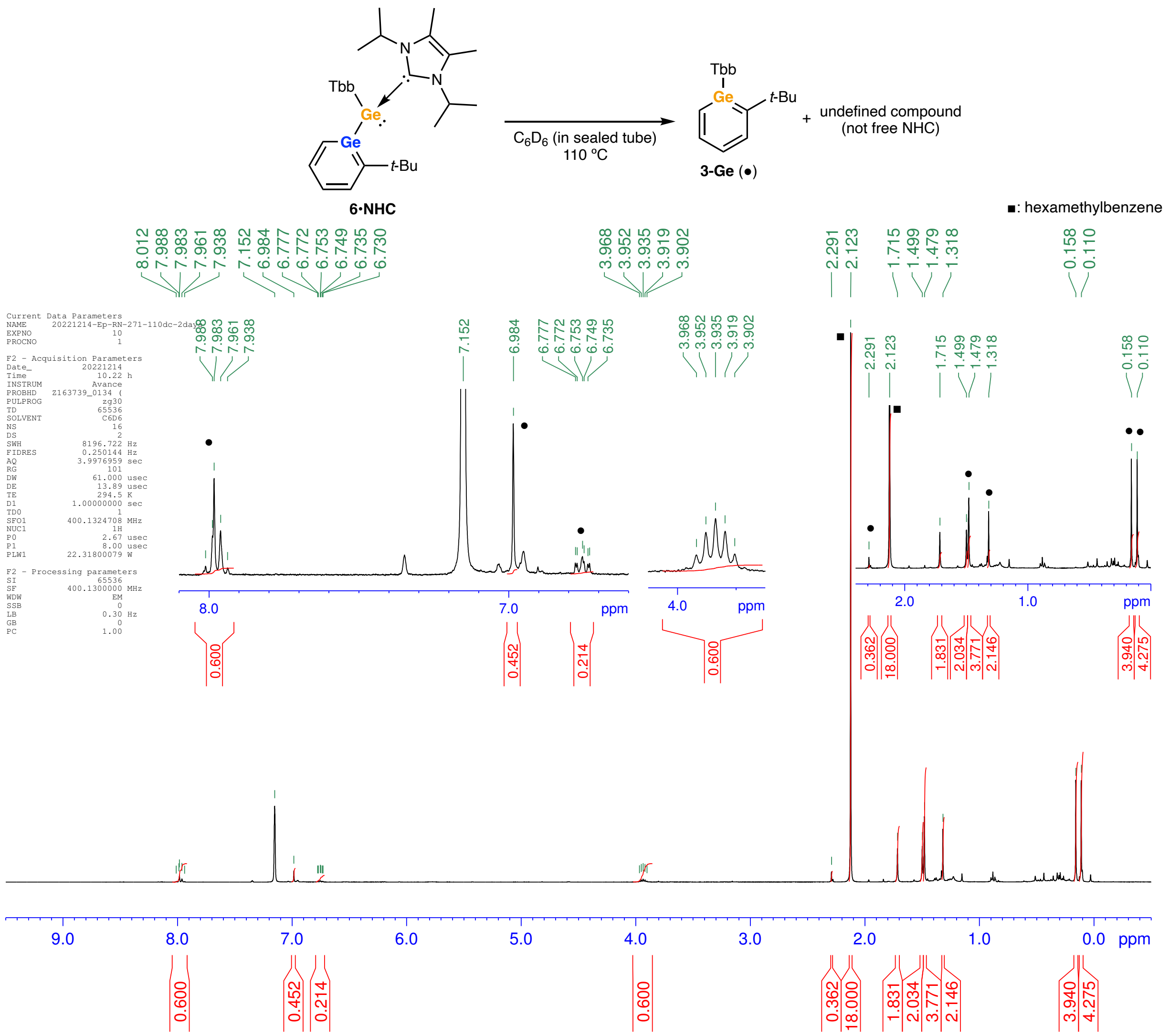

Supplementary Fig. 34. ^{1}H NMR spectrum of the mixture after heating C_6D_6 solution of **6·NHC** at 110 °C for 2 days (C_6D_6, 400 MHz, r.t.).

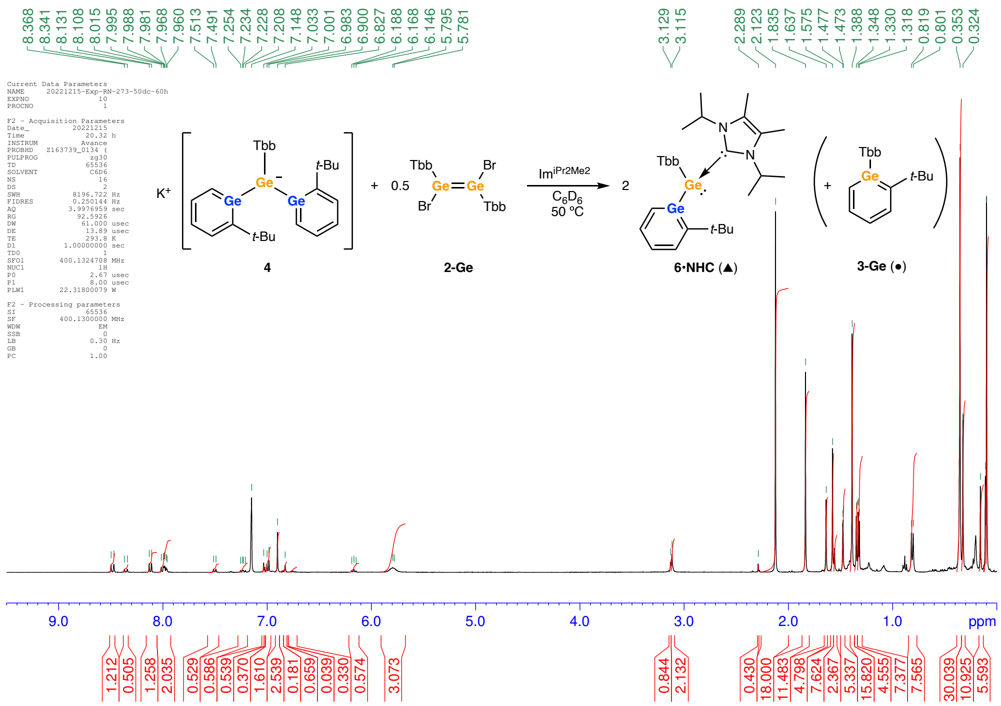

Supplementary Fig. 35. ^{1}H NMR spectrum of the mixture after the reaction of **4** with **2-Ge** in the presence of Im^{iPr2Me2} (C_6D_6, 400 MHz, r.t.).

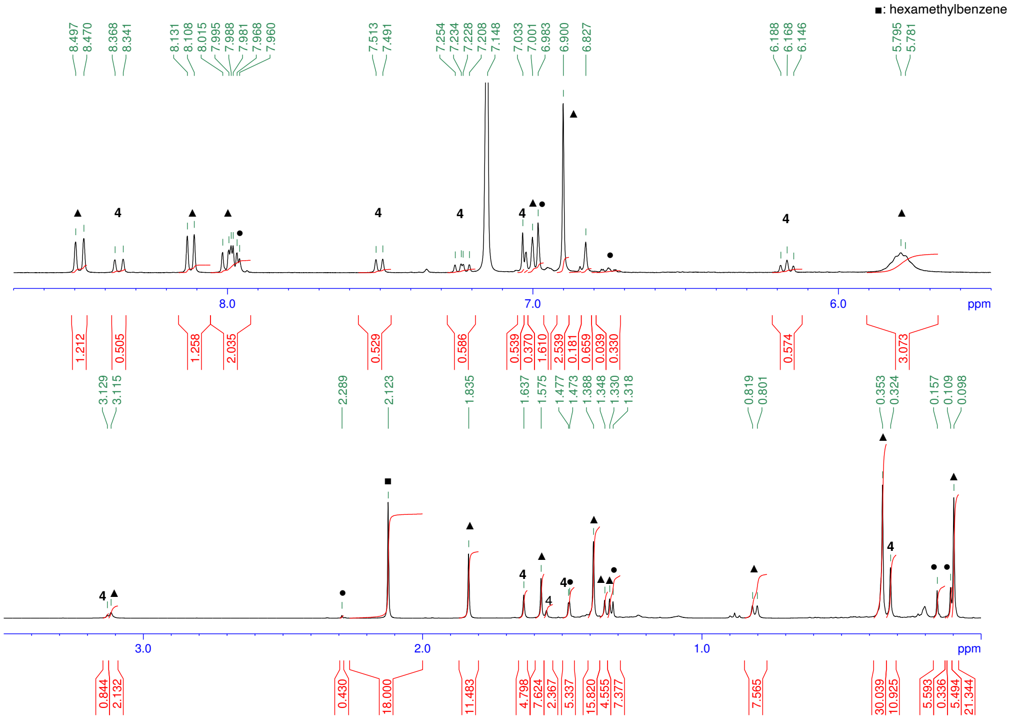

Supplementary Fig. 36. Magnified ^{1}H NMR spectrum of the mixture after the reaction of **4** with **2-Ge** in the presence of Im^{iPr2Me2} (C_6D_6, 400 MHz, r.t.).

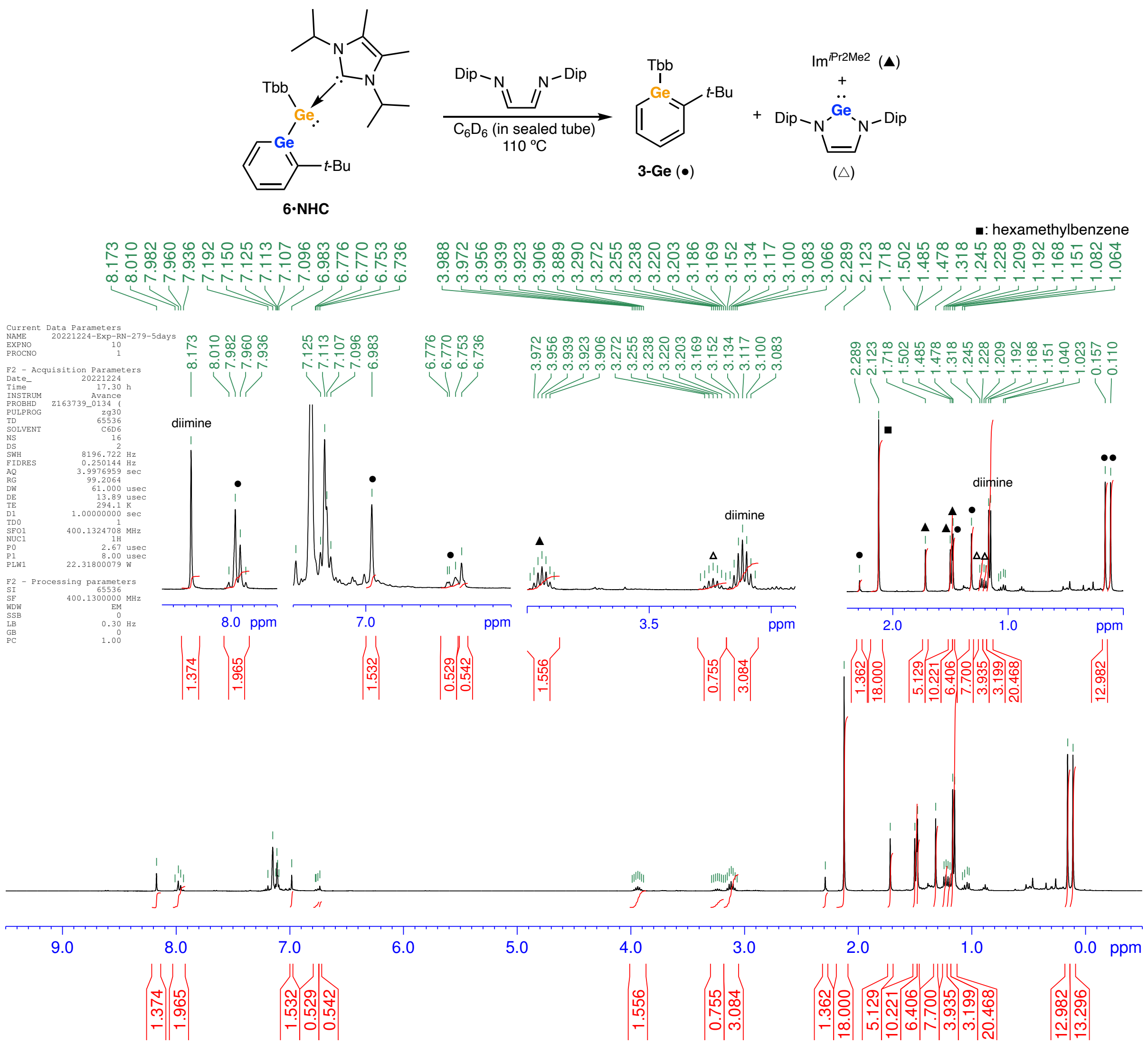

Supplementary Fig. 37. ^{1}H NMR spectrum of the mixture after heating C_6D_6 solution of **6·NHC** in the presence of diimine at 110 °C for 5 days (C_6D_6, 400 MHz, r.t.).

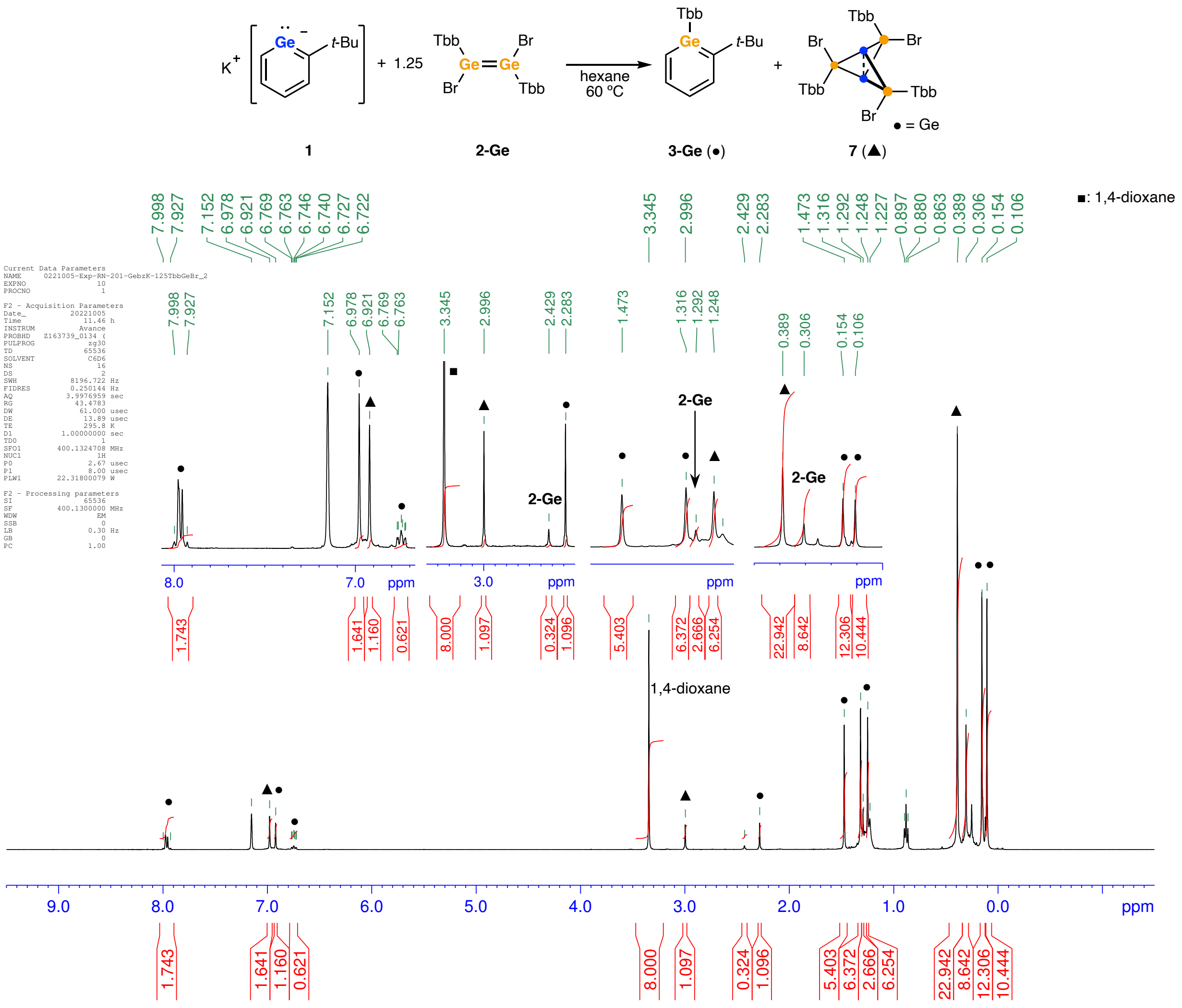

Supplementary Fig. 38. ^{1}H NMR spectrum of the mixture after the reaction of **1** with 1.25 eq. of **2-Ge** (C_6D_6, 400 MHz, r.t.).

6. Computational details

All geometry optimizations and single point calculations were carried out using Gaussian 16 software-suite.[10] All geometries of **6** and all intermediates were optimized at B3LYP-D3/6-31+G(2df,p) level of theory. Natural bonding orbital (NBO) analysis were performed using NBO 7.0 program[11] and calculated at B3LYP-D3/6-311+G(2df,p) level of theory.

Potential energy surface exploration of Gebzl(H)Ge:, Gebzl = 1-germabenzenyl (**6a**) was performed using GRRM17 program[12] and calculated with ADDF method and First-Only option at B3LYP/6-31G level of theory. Isomerization pathway of **6** including the molecular geometries for the transition states were first estimated by Reaction plus Pro 2 software package,[13] based on the nudged elastic band (NEB) method,[14,15] and were subsequently re-optimized using Gaussian 16 software package. All Kohn-Sham orbital, natural bonding orbital, and molecular structures were visualized using Avogadro[16] and POV-Ray softwares.[17] For intrinsic bonding orbital (IBO) analysis, single point energy calculations were first performed by ORCA software[18,19] at B3LYP-D3/def2-TZVP level of theory (D3BJ keyword was used for dispersion correction) using optimized geometry obtained by Gaussian. Then, generated .gbw file was read by IboView to calculate IBOs.[20,21]

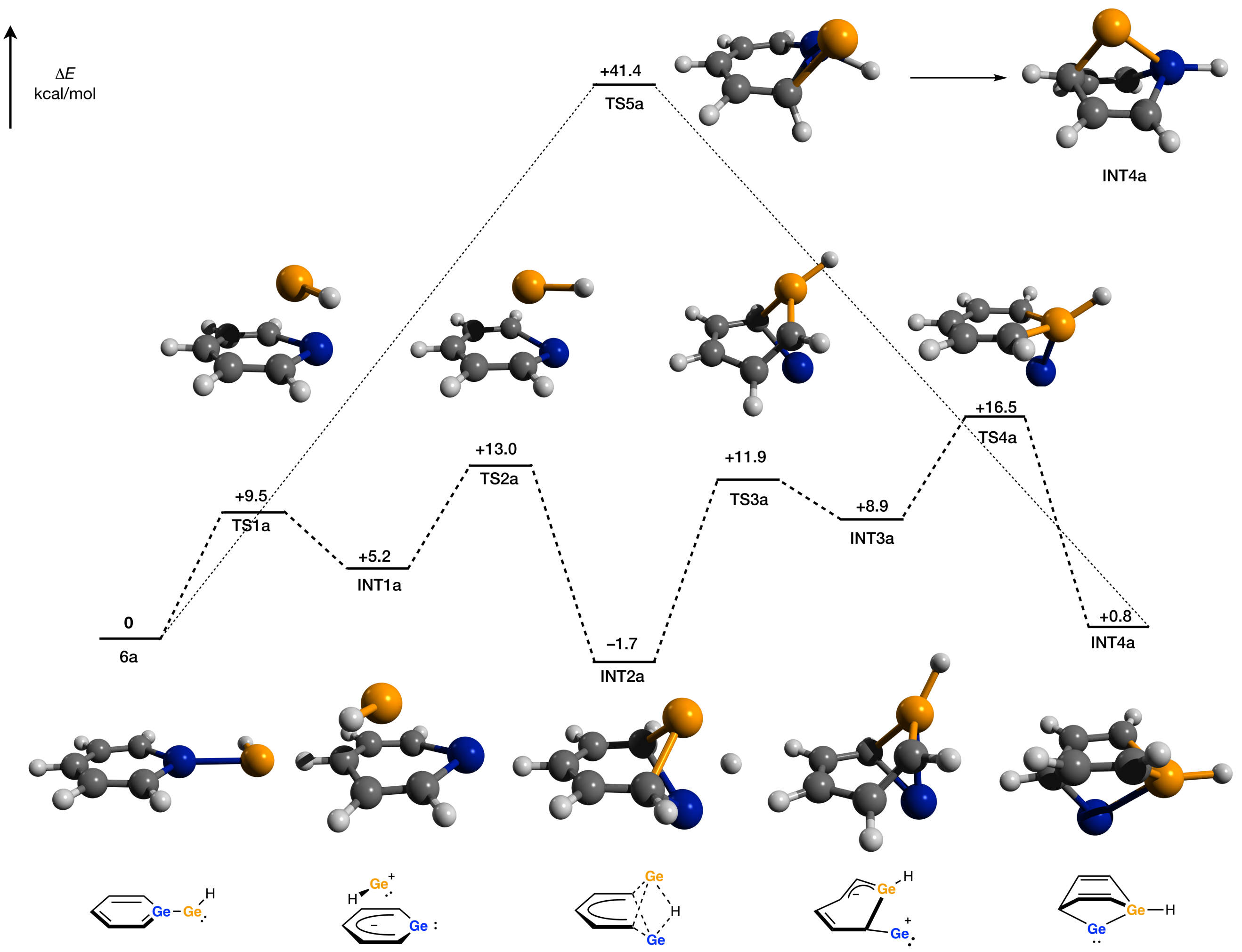

Supplementary Fig. 39. Energy diagram of the model compound **6a** by GRRM calculations at ADDF-B3LYP/6-31G level of theory.

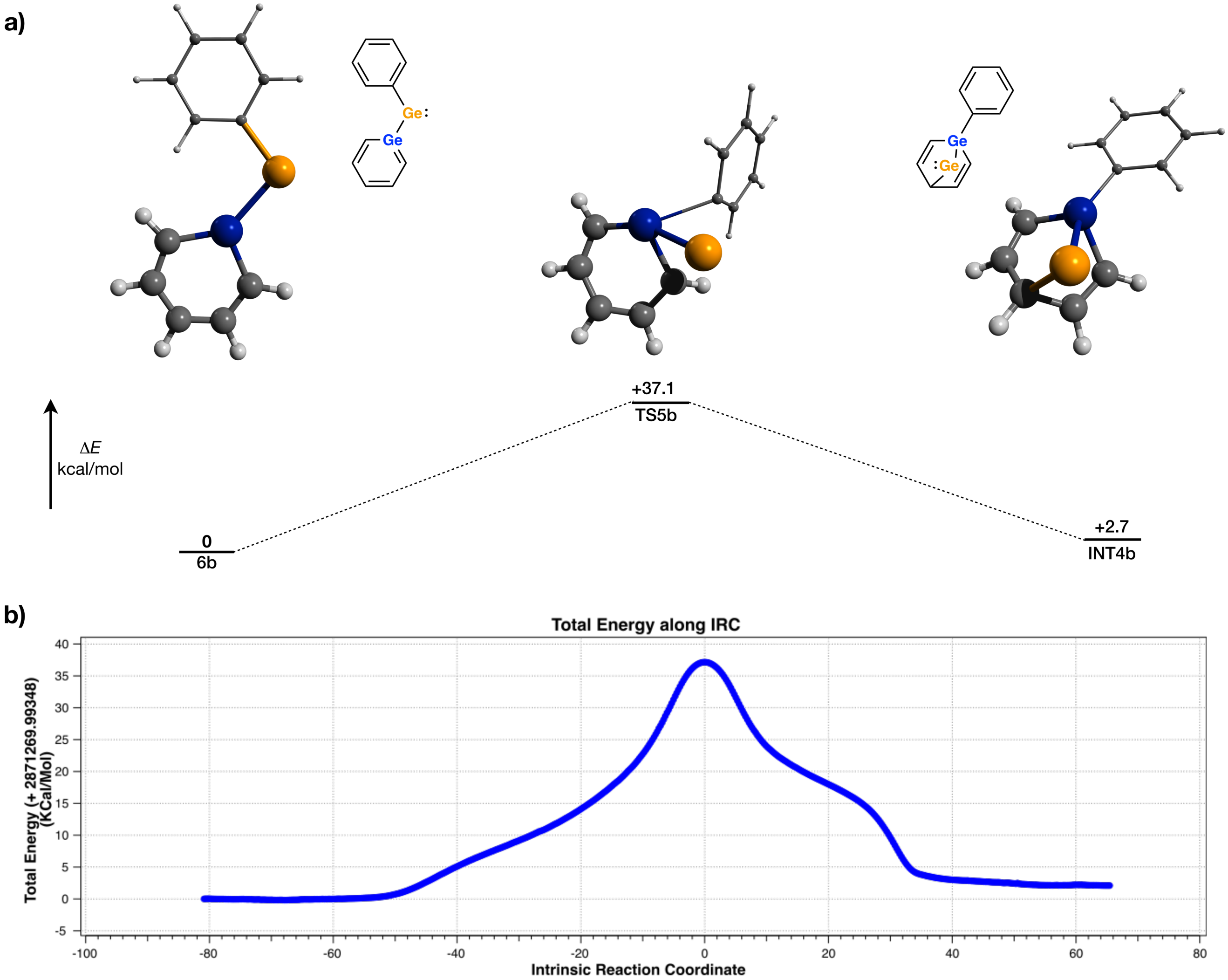

Supplementary Fig. 40. a) Energy diagram of model compound **6b** to give **INT4b** *via* one step pathway (Ph group transfer). B3LYP-D3/6-31+G(2df,p) level of theory was employed. b) Energy diagram of **6b** obtained from the IRC calculations of **TS5b**.

6

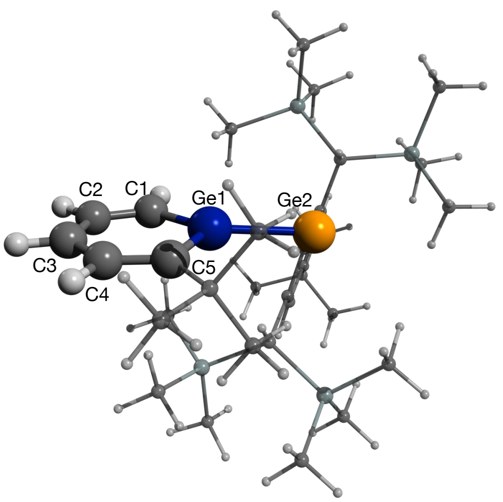

INT1

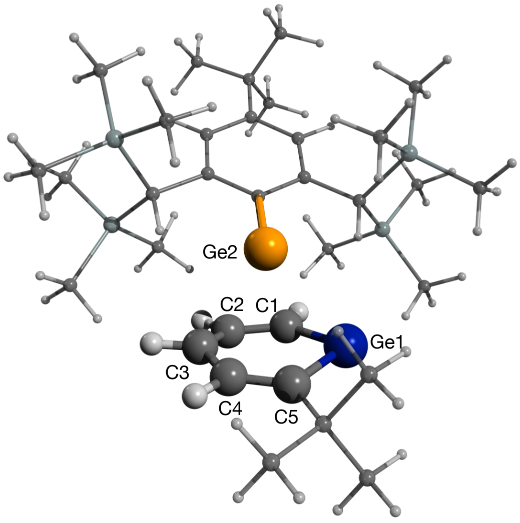

INT2

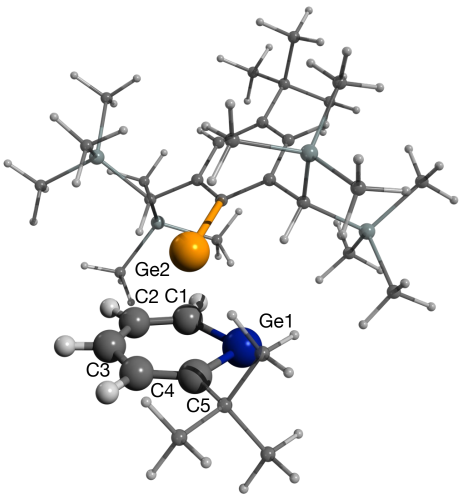

INT3

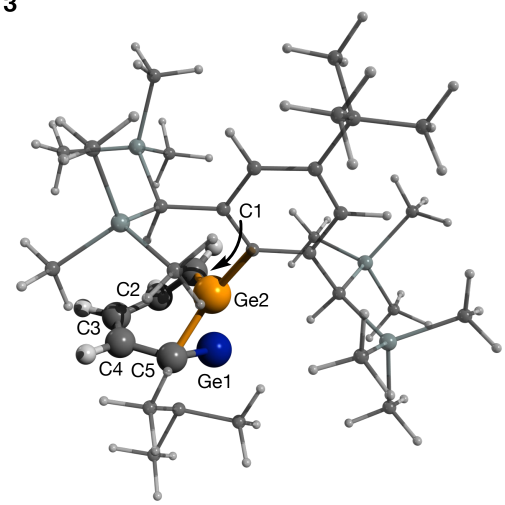

INT4

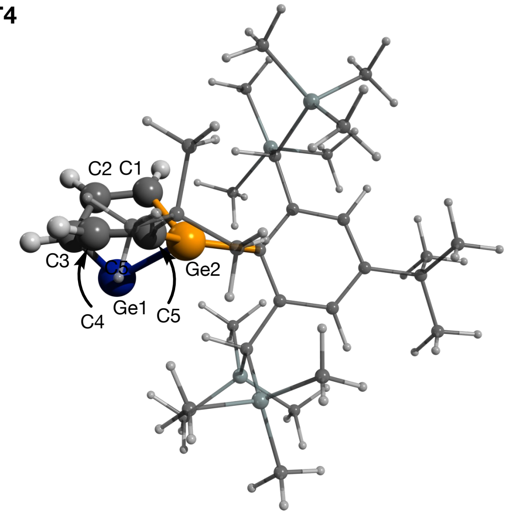

Supplementary Fig. 41. Optimized structures of **6** and intermediates at B3LYP-D3/6-31+G(2df,p) level of theory.

Supplementary Table 9. Selected bond lengths (Å) and angles (°) for **6**.

	distance (Å)		angle (°)
Ge1–C1	1.856	C1–Ge1–C5	104.9
C1–C2	1.390	C2–C1–Ge1	119.7
C2–C3	1.398	C3–C2–C1	124.9
C3–C4	1.406	C4–C3–C2	126.3
C4–C5	1.393	C5–C4-C3	127.9
C5–Ge1	1.882	Ge1–C5–C4	116.3
Ge1–Ge2	2.465	Ge1–Ge2–C6	98.7

Supplementary Table 10. Selected bond lengths (Å) and angles (°) for **INT1**.

	distance (Å)		angle (°)
Ge1–C1	1.941	C1–Ge1–C5	96.0
C1–C2	1.410	C2–C1–Ge1	125.1
C2–C3	1.405	C3–C2–C1	122.2
C3–C4	1.432	C4–C3–C2	125.3
C4–C5	1.368	C5–C4–C3	126.7
C5–Ge1	1.968	Ge1–C5–C4	121.7
Ge1–Ge2	2.868		
Ge2–C1	2.304		
Ge2–C2	2.415		
Ge3–C3	2.663		
Ge2–C4	3.149		
Ge2–C5	3.291		

Supplementary Table 11. Selected bond lengths (Å) and angles (°) for **INT2**.

	distance (Å)		angle (°)
Ge1–C1	1.925	C1–Ge1–C5	95.8
C1–C2	1.410	C2–C1–Ge1	125.6
C2–C3	1.399	C3–C2–C1	123.5
C3–C4	1.410	C4–C3–C2	125.6
C4–C5	1.385	C5–C4–C3	126.5
C5–Ge1	1.956	Ge1–C5–C4	122.9
Ge1–Ge2	2.787		
Ge2–C1	2.348		
Ge2–C2	2.656		
Ge3–C3	3.065		
Ge2–C4	3.292		
Ge2–C5	3.154		

Supplementary Table 12. Selected bond lengths (Å) and angles (°) for **INT3**.

	distance (Å)		angle (°)
Ge2–C1	1.875	C1–Ge2–C5	93.1
C1–C2	1.433	C2–C1–Ge2	118.0
C2–C3	1.472	C3–C2–C1	119.6
C3–C4	1.347	C4–C3–C2	118.4
C4–C5	1.483	C5–C4–C3	118.1
C5–Ge2	1.971	Ge2–C5–C4	108.2
Ge1–Ge2	2.511	Ge2–C1–C5	92.58
Ge1–C1	2.308	Ge1–C5–Ge2	109.13
Ge1–C2	2.249	C5–Ge2–Ge1	57.75
Ge1–C3	2.940	Ge2–Ge1–C5	50.55
Ge1–C4	2.929	Ge2–C5–Ge1	74.70
Ge1–C5	2.096		

Supplementary Table 13. Selected bond lengths (Å) and angles (°) for **INT4**.

	distance (Å)		angle (°)
Ge2–C1	1.951	C1–Ge2–C5	99.9
C1–C2	1.386	C2–C1–Ge2	107.2
C2–C3	1.486	C3–C2–C1	123.4
C3–C4	1.493	C4–C3–C2	117.3
C4–C5	1.343	C5–C4–C3	118.1
C5–Ge2	1.957	Ge2–C5–C4	110.4
Ge1–Ge2	2.482	Ge2–Ge1–C3	75.3
Ge1–C1	2.455		
Ge1–C2	2.258		
Ge1–C3	2.104		
Ge1–C4	3.113		
Ge1–C5	3.440		

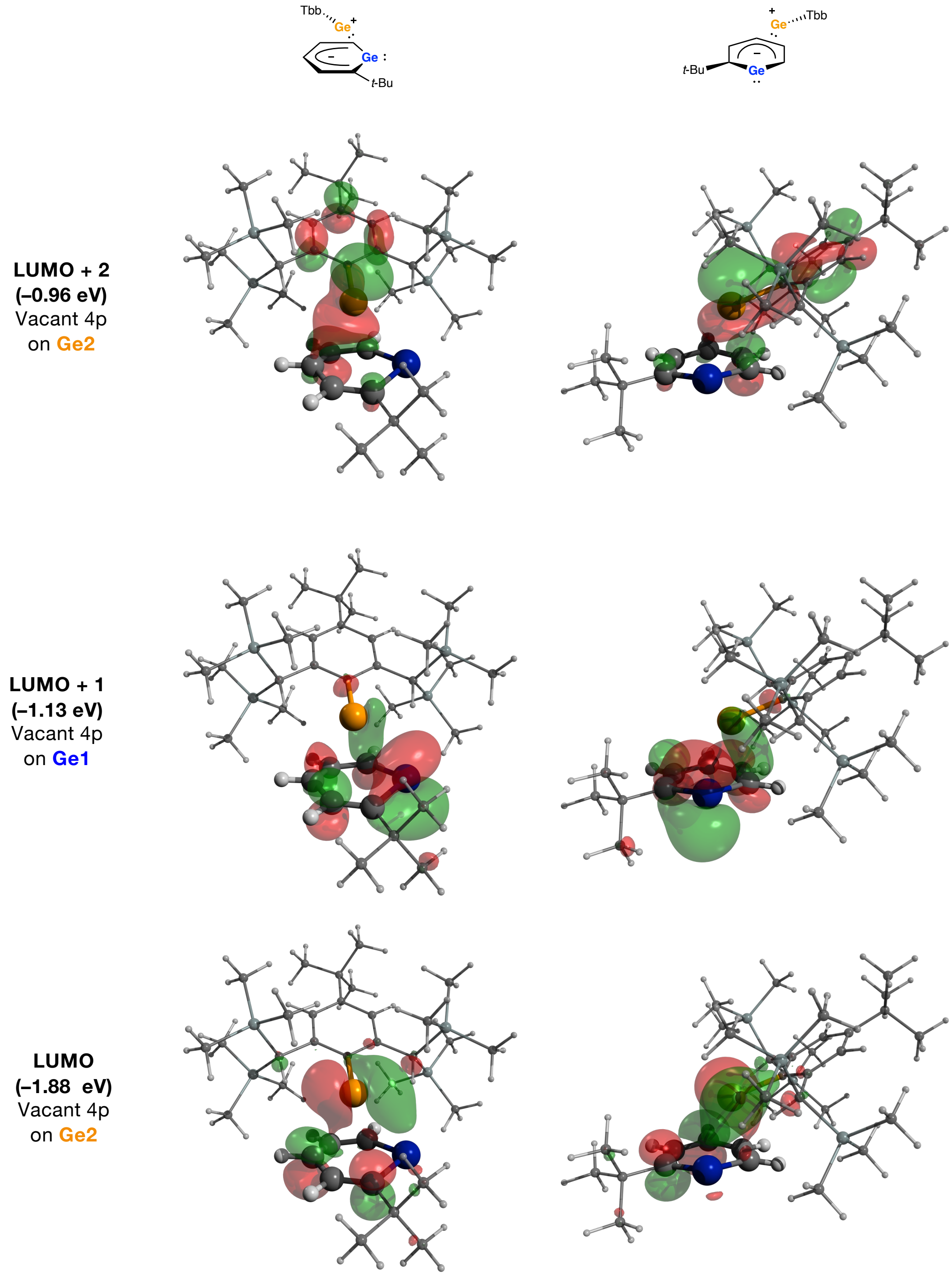

Supplementary Fig. 42. Selected Kohn-Sham orbitals of **INT1** at B3LYP-D3/6-31+G(2df,p) level of theory.

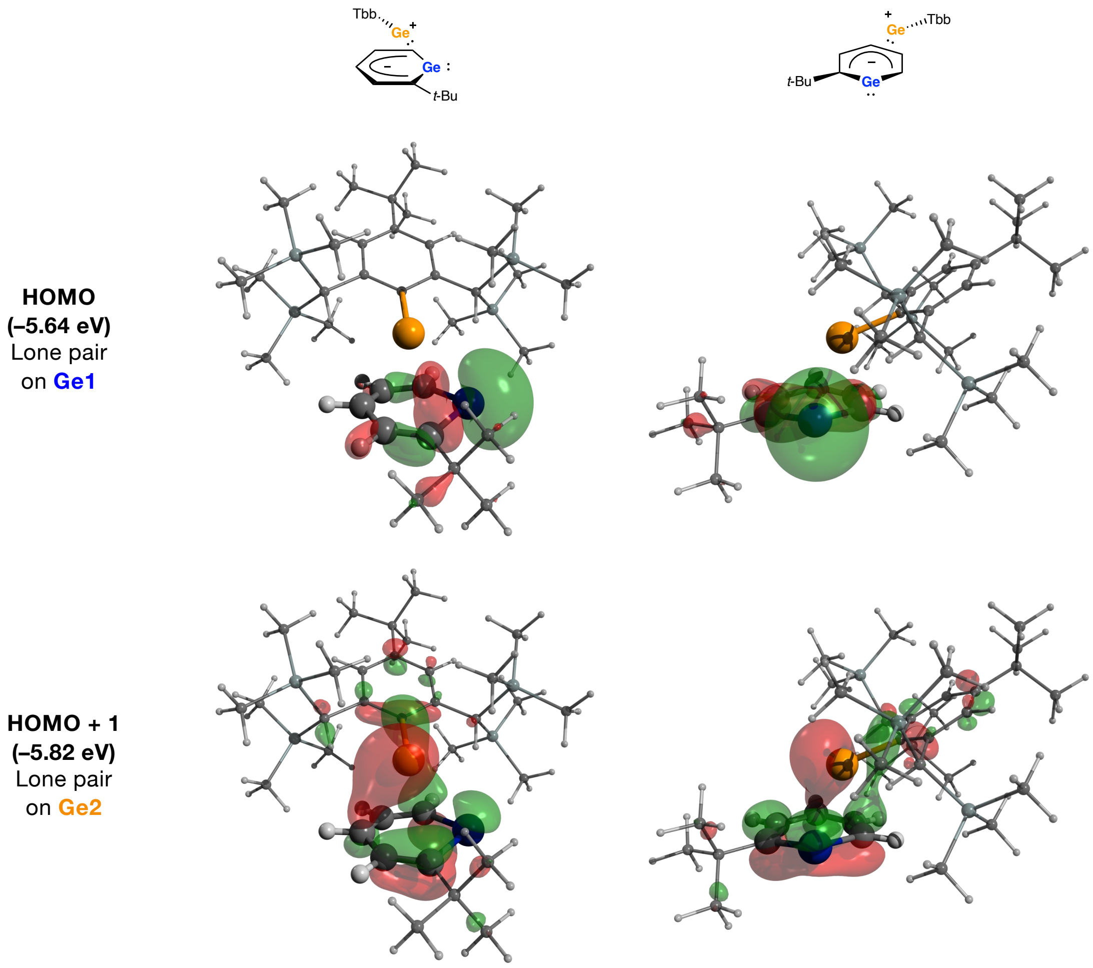

Supplementary Fig. 43. Selected Kohn-Sham orbitals of **INT1** at B3LYP-D3/6-31+G(2df,p) level of theory.

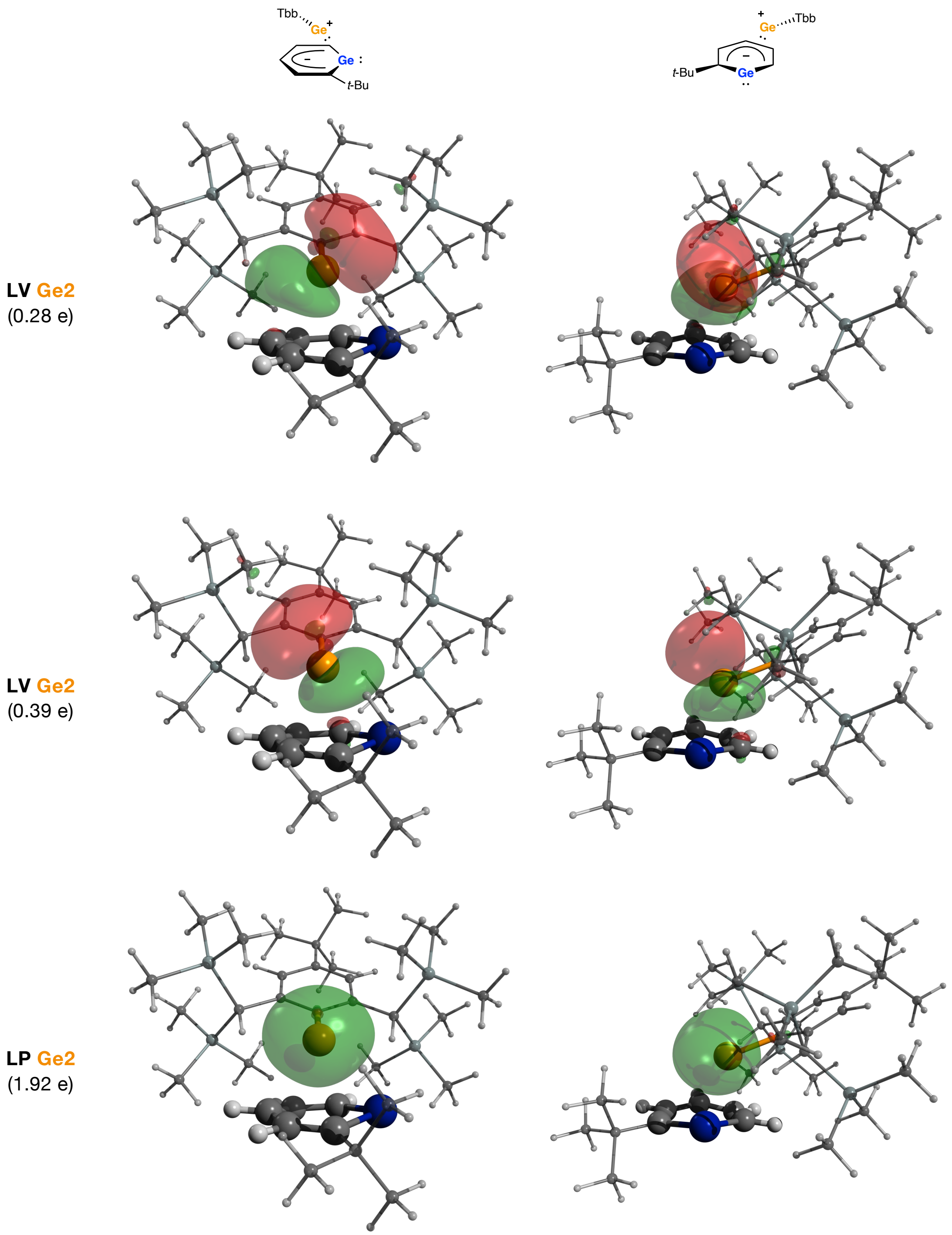

Supplementary Fig. 44. Selected natural bonding orbitals of **INT1** at B3LYP-D3/6-311+G(2df,p) level of theory.

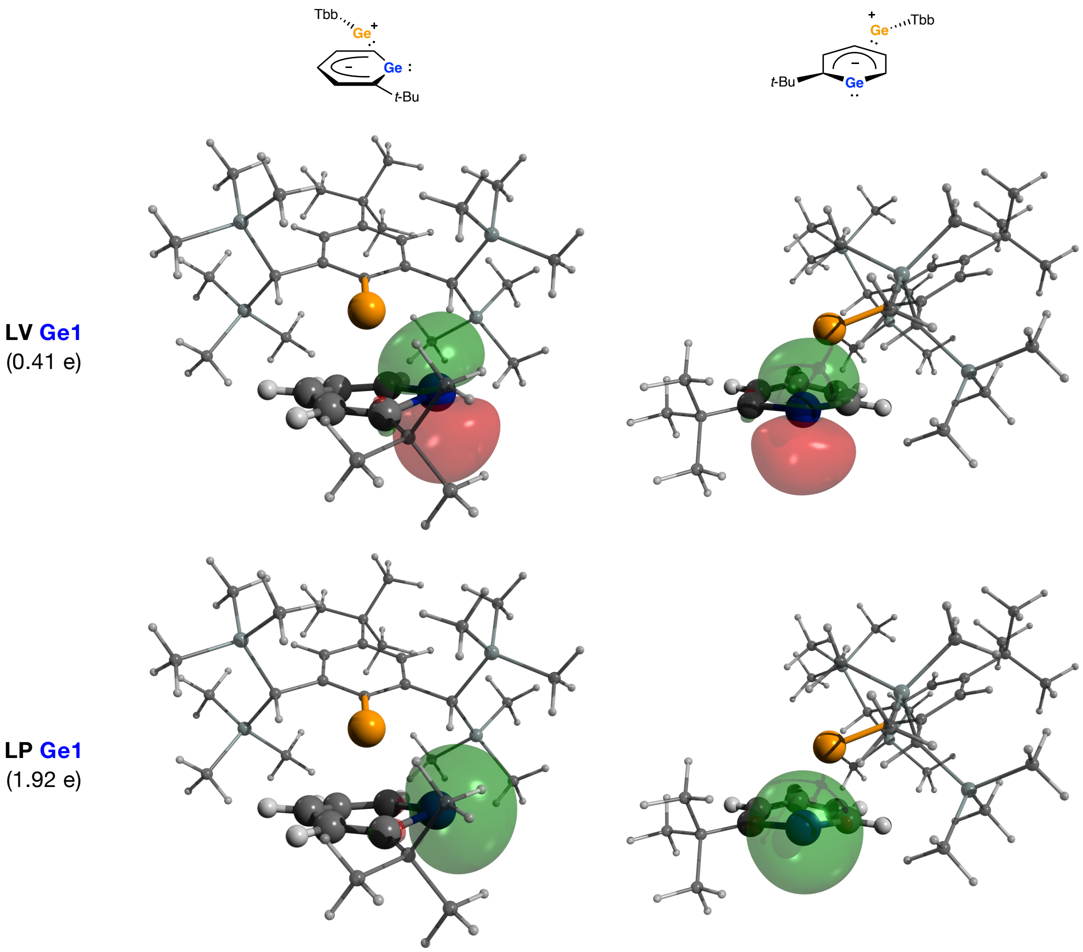

Supplementary Fig. 45. Selected natural bonding orbitals of **INT1** at B3LYP-D3/6-311+G(2df,p) level of theory.

LUMO + 2
(–0.66 eV)
Vacant 4p
on **Ge1**

LUMO + 1
(–1.23 eV)
Vacant 4p
on **Ge2**

LUMO
(–2.53 eV)
Vacant 4p
on **Ge2**

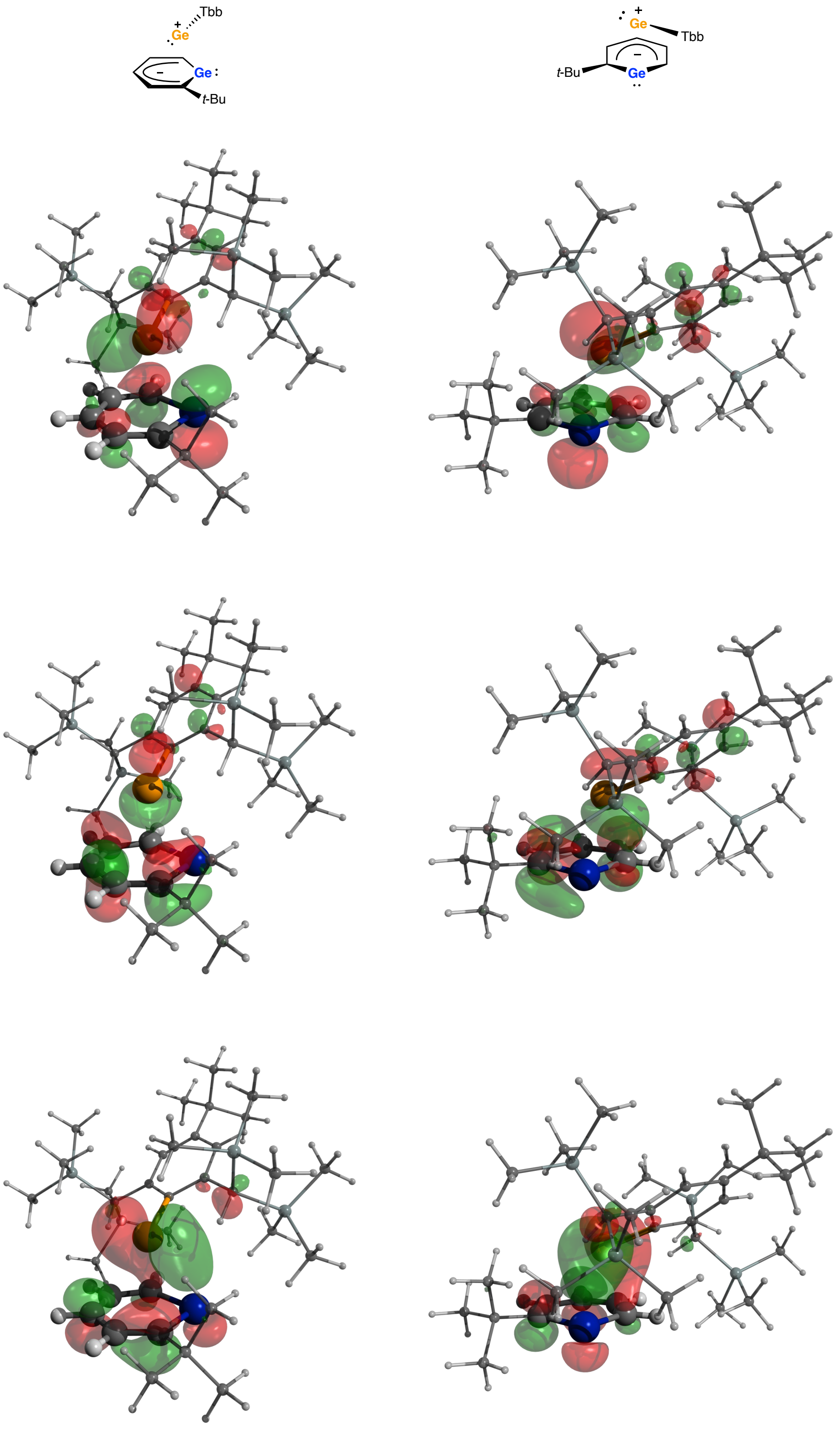

Supplementary Fig. 46. Selected Kohn-Sham orbitals of **INT2** at B3LYP-D3/6-31+G(2df,p) level of theory.

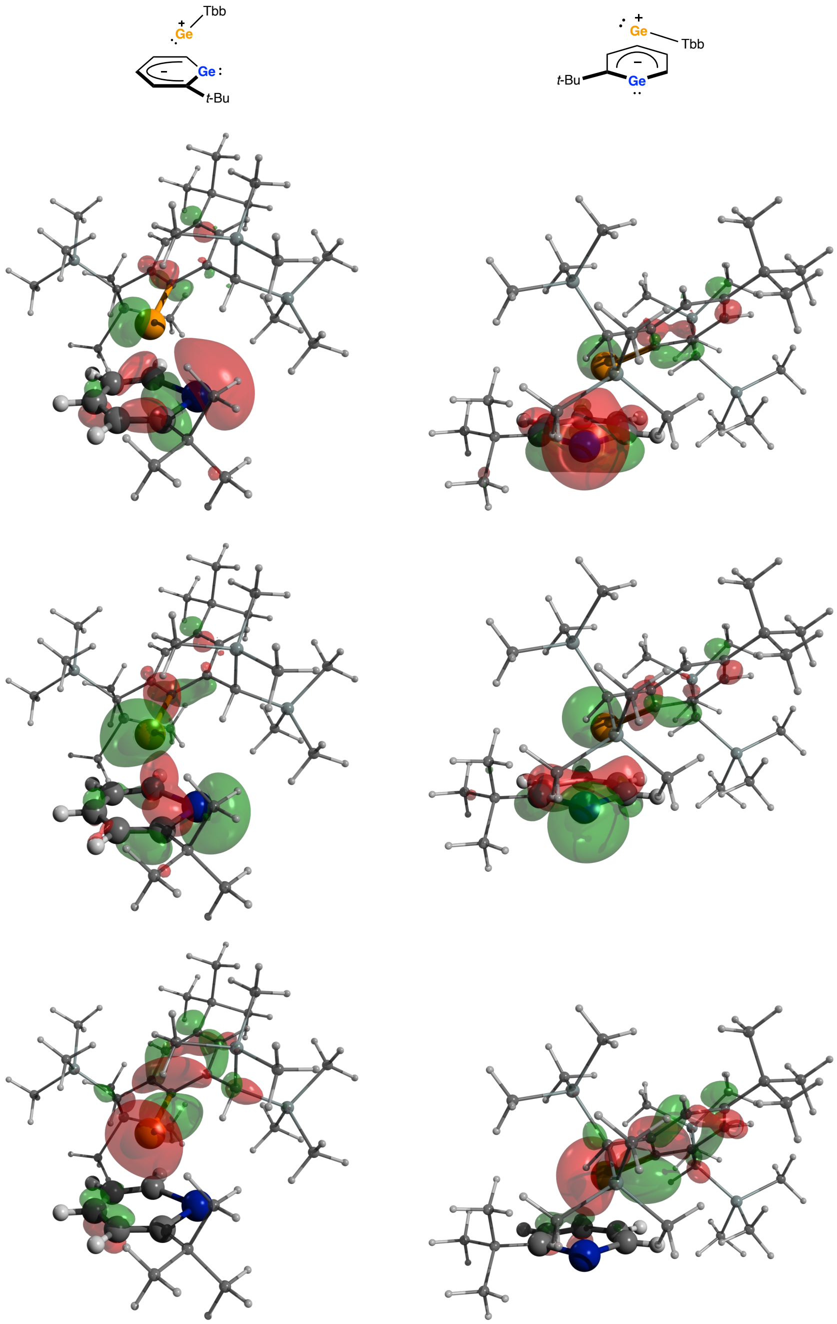

HOMO
(–5.61 eV)
Lone pair
on **Ge1**

HOMO – 1
(–5.62 eV)
Lone pair
on
Ge1 and **Ge2**

HOMO – 3
(–6.43 eV)
Lone pair
on **Ge2**

Supplementary Fig. 47. Selected Kohn-Sham orbitals of **INT2** at B3LYP-D3/6-31+G(2df,p) level of theory.

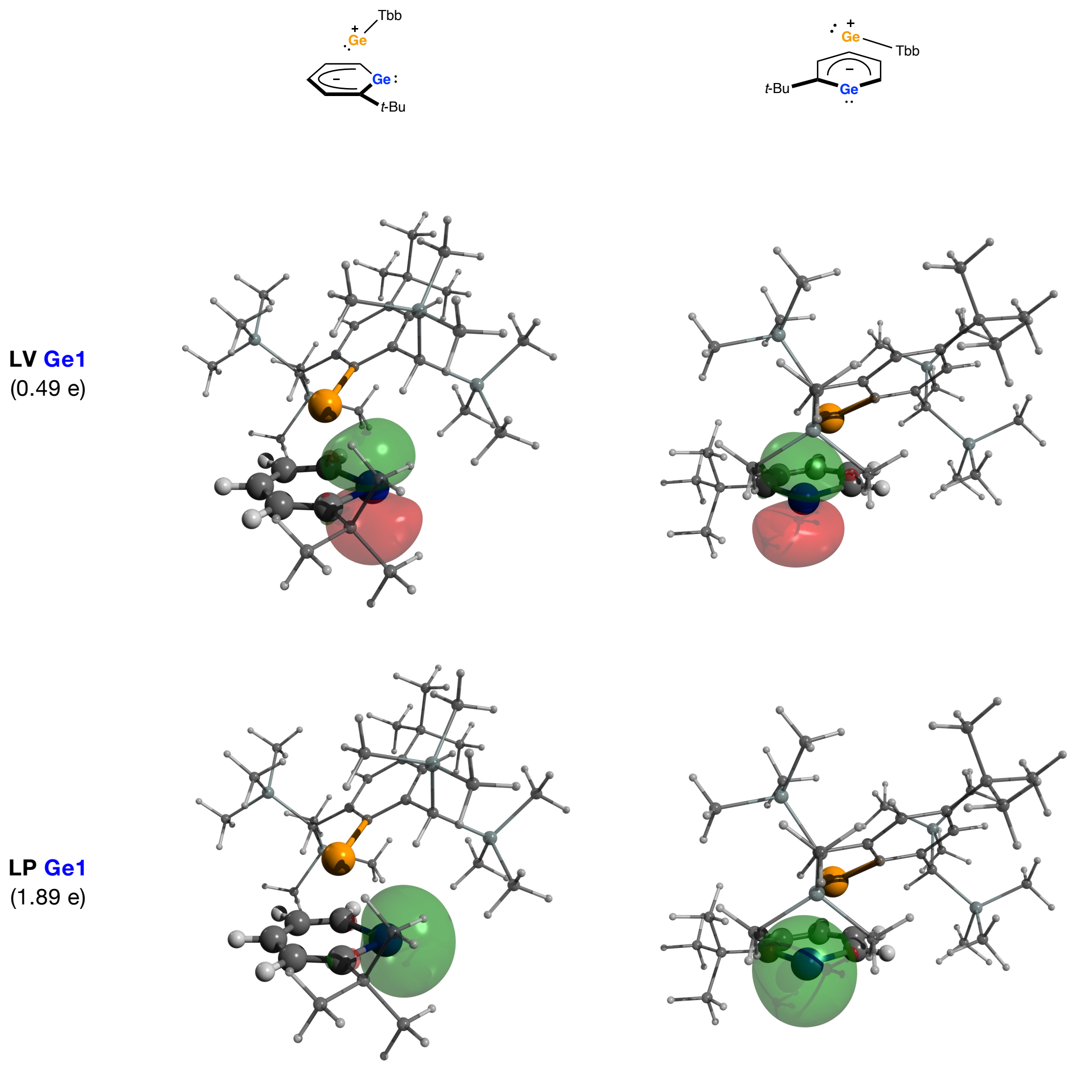

Supplementary Fig. 48. Selected natural bonding orbitals of **INT2** at B3LYP-D3/6-311+G(2df,p) level of theory.

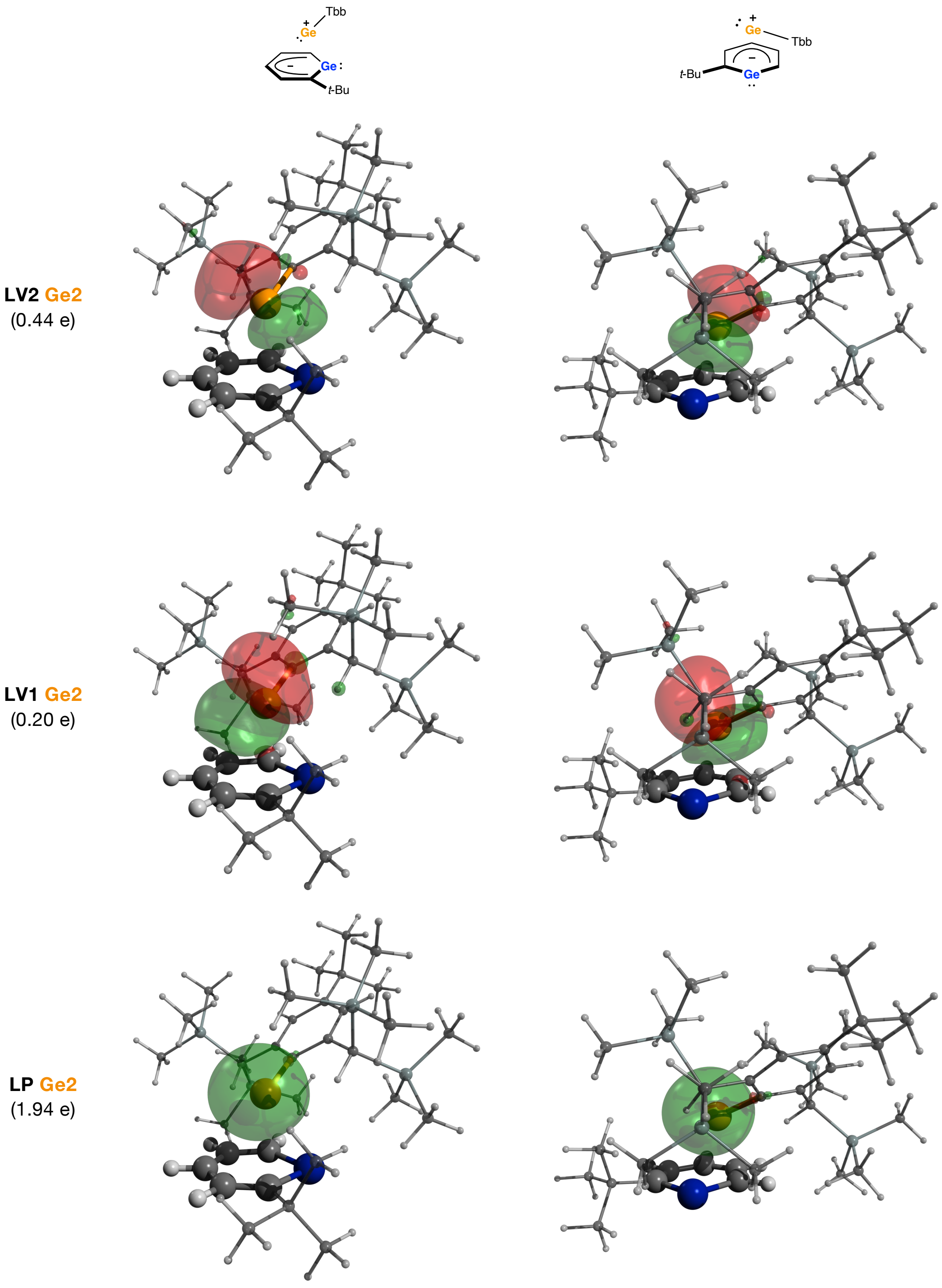

Supplementary Fig. 49. Selected natural bonding orbitals of **INT2** at B3LYP-D3/6-311+G(2df,p) level of theory.

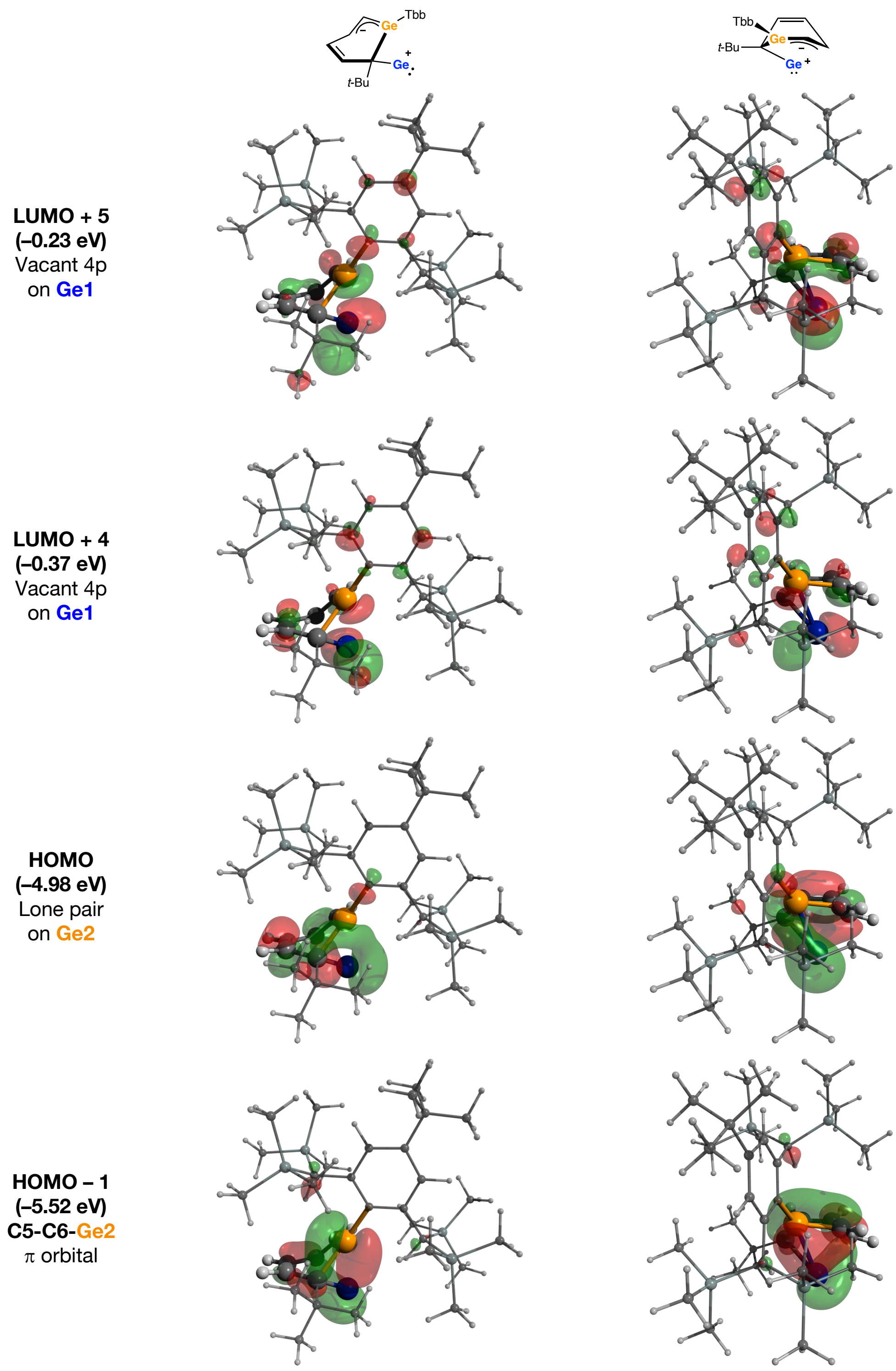

Supplementary Fig. 50. Selected Kohn-Sham orbitals of **INT3** at B3LYP-D3/6-31+G(2df,p) level of theory.

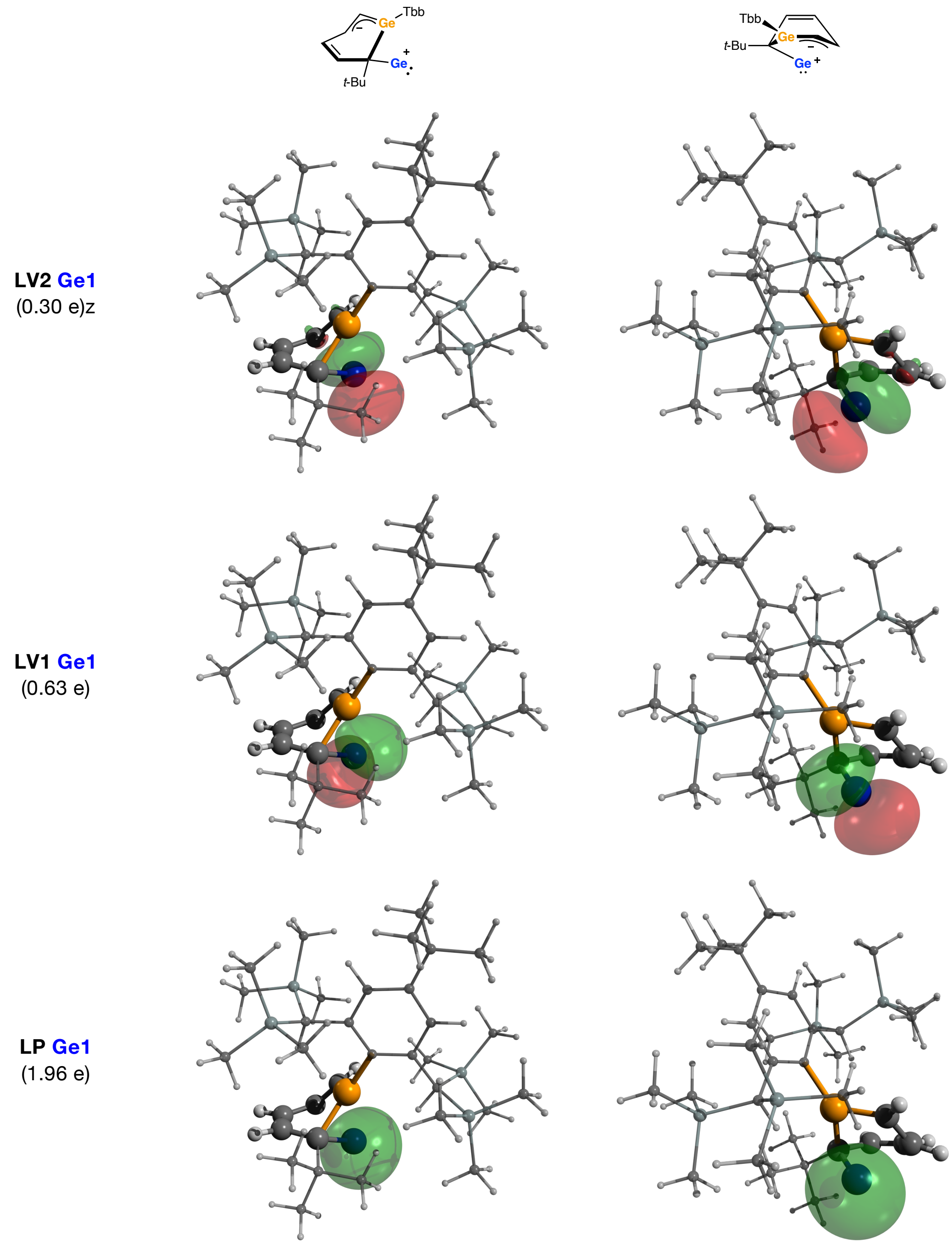

Supplementary Fig. 51. Selected natural bonding orbitals of **INT3** at B3LYP-D3/6-311+G(2df,p) level of theory.

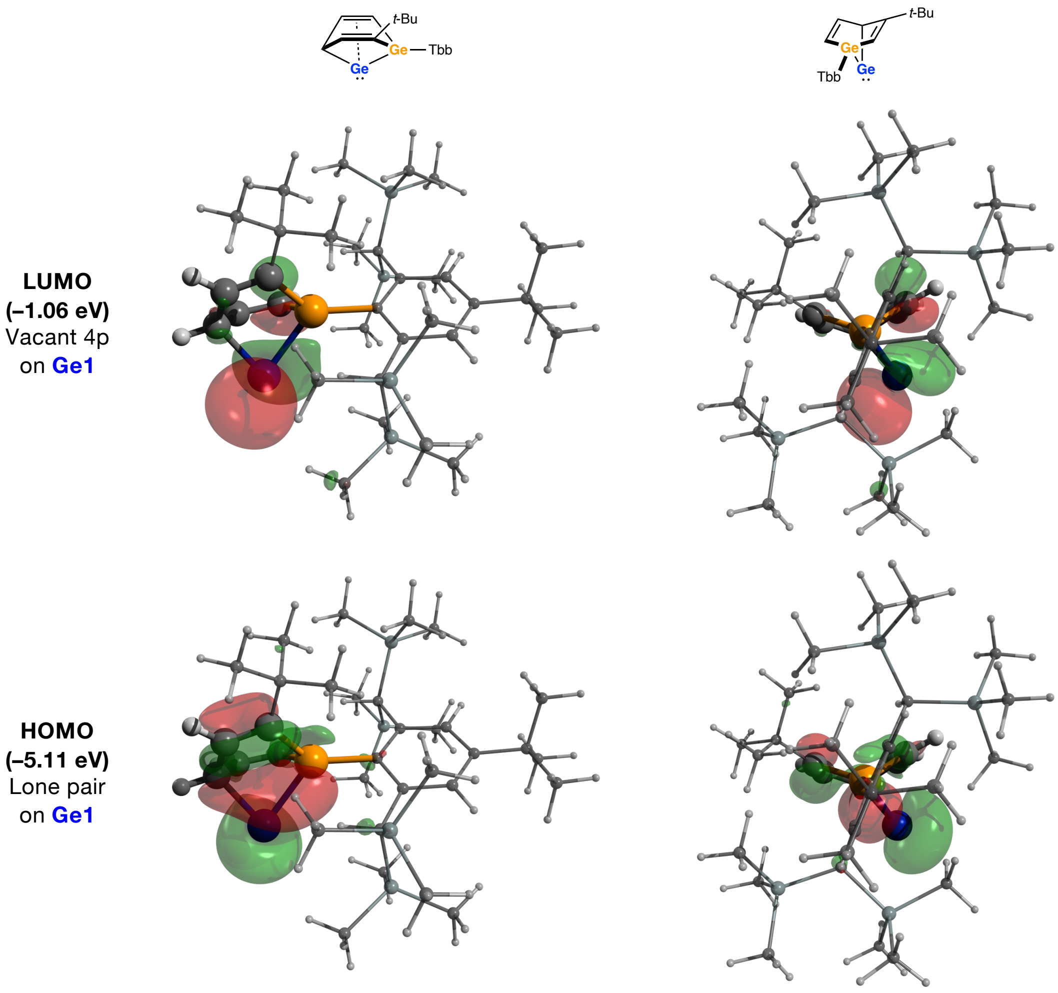

Supplementary Fig. 52. Selected Kohn-Sham orbitals of **INT4** at B3LYP-D3/6-31+G(2df,p) level of theory.

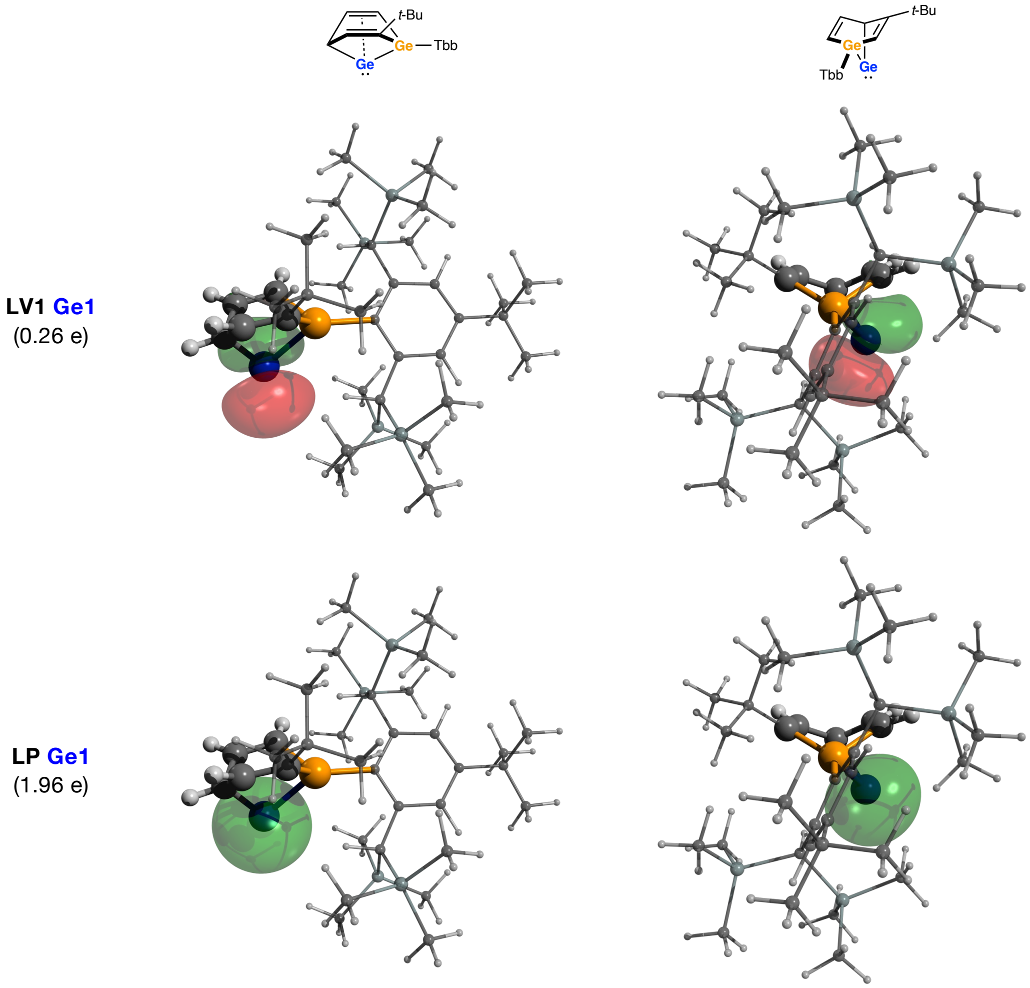

Supplementary Fig. 53. Selected natural bonding orbitals of **INT4** at B3LYP-D3/6-311+G(2df,p) level of theory.

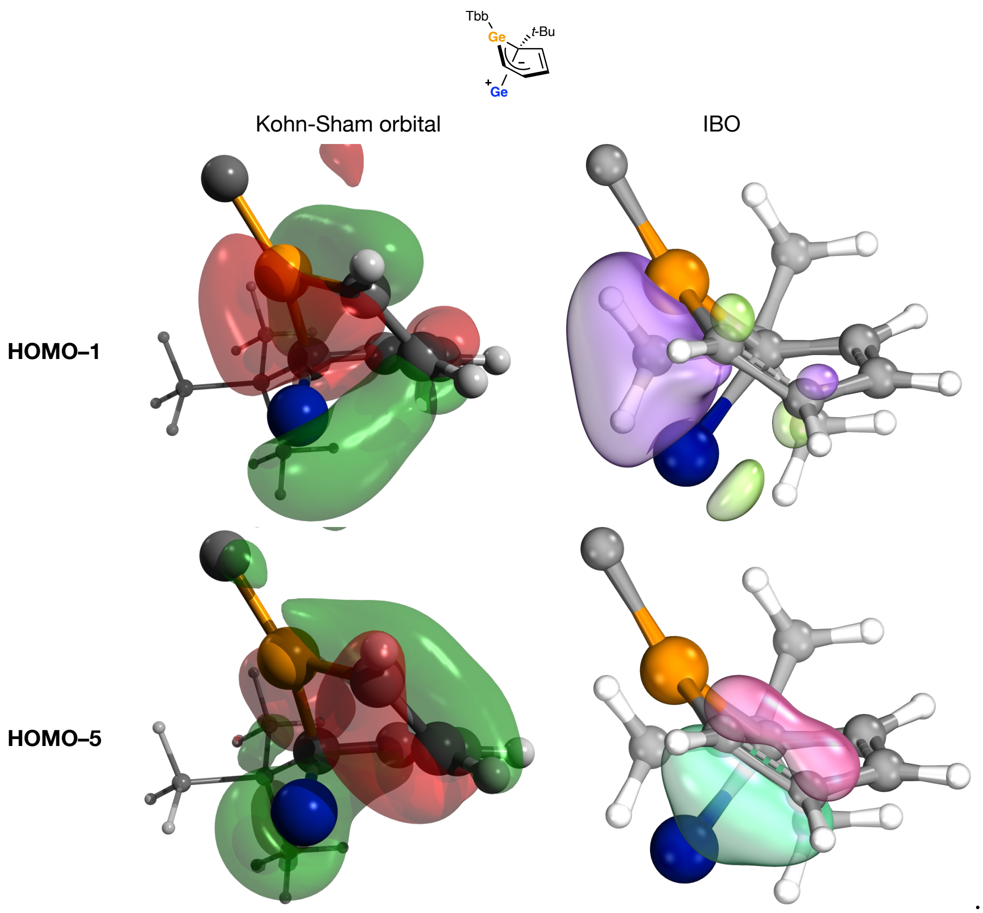

Supplementary Fig. 54. Kohn-Sham orbitals and IBOs of **INT3** exhibit the electron delocalization among Ge2–C1–C2–Ge1 moiety.

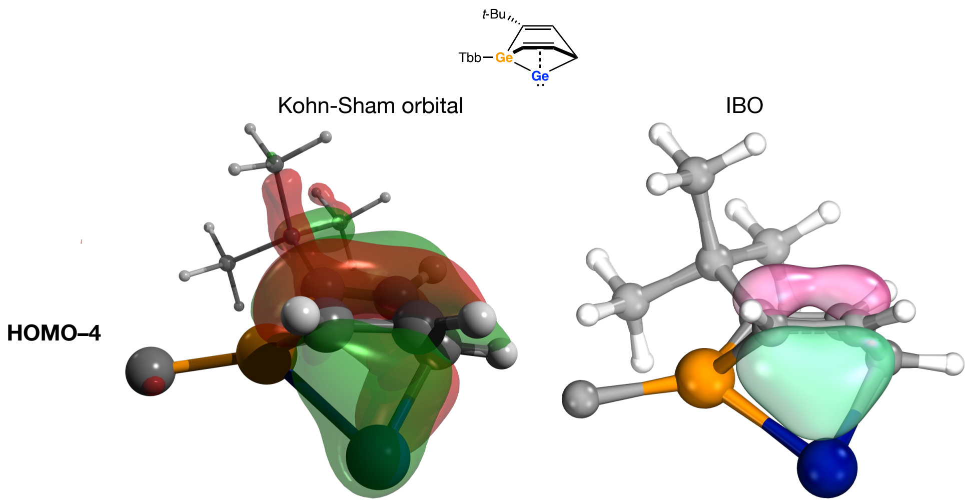

Supplementary Fig. 55. Kohn-Sham orbitals and IBOs of **INT4** exhibit the electron delocalization among C1–C2–Ge1 moiety.

7. References

1. Pangborn, A B., Giardello, M A., Grubbs, R H., Rosen, R K. & Timmers, F J. Safe and convenient procedure for solvent purification. *Organometallics* **15,** 1518-1520 (1996).
2. Tang, P., Wang, W. & Ritter, T. Deoxyfluorination of phenols. *J. Am. Chem. Soc.* **133,** 11482-11484 (2011).
3. Kuhn, N. & Kratz, T. Synthesis of imidazol-2-ylidenes by reduction of imidazole-2(3*H*)-thiones. *Synthesis* **1993,** 561-562 (1993).
4. Mizuhata, Y., Fujimori, S., Sasamori, T. & Tokitoh, N. Germabenzenylpotassium: A germanium analogue of a phenyl anion. *Angew. Chem. Int. Ed.* **56,** 4588-4592 (2017).
5. Sugahara, T., Guo, J D., Sasamori, T., Karatsu, Y., Furukawa, Y., Ferao, A E., Nagase, S. & Tokitoh, N. Reaction of a stable digermyne with acetylenes: Synthesis of a 1,2-digermabenzene and a 1,4-digermabarrelene. *Bull. Chem. Soc. Jpn.* **89,** 1375-1384 (2016).
6. Agou, T., Hayakawa, N., Sasamori, T., Matsuo, T., Hashizume, D. & Tokitoh, N. Reactions of diaryldibromodisilenes with *N*-heterocyclic carbenes: Formation of formal bis-NHC adducts of silyliumylidene cations. *Chem. Eur. J.* **20,** 9246-9249 (2014).
7. Baker, R J., Jones, C., Mills, D P., Pierce, G A. & Waugh, M. Investigations into the preparation of groups 13–15 *N*-heterocyclic carbene analogues. *Inorg. Chim. Acta* **361,** 427-435 (2008).
8. Sheldrick, G. SHELXT - integrated space-group and crystal-structure determination. *Acta Cryst. A* **71,** 3-8 (2015).
9. Sheldrick, G. Crystal structure refinement with SHELXL. *Acta Cryst. C* **71,** 3-8 (2015).
10. Frisch, M J., Trucks, G W., Schlegel, H B., Scuseria, G E., Robb, M A., Cheeseman, J R., Scalmani, G., Barone, V., Petersson, G A., Nakatsuji, H., Li, X., Caricato, M., Marenich, A V., Bloino, J., Janesko, B G., Gomperts, R., Mennucci, B., Hratchian, H P., Ortiz, J V., Izmaylov, A F., Sonnenberg, J L., Williams., Ding, F., Lipparini, F., Egidi, F., Goings, J., Peng, B., Petrone, A., Henderson, T., Ranasinghe, D., Zakrzewski, V G., Gao, J., Rega, N., Zheng, G., Liang, W., Hada, M., Ehara, M., Toyota, K., Fukuda, R., Hasegawa, J., Ishida, M., Nakajima, T., Honda, Y., Kitao, O., Nakai, H., Vreven, T., Throssell, K., Montgomery Jr., J A., Peralta, J E., Ogliaro, F., Bearpark, M J., Heyd, J J., Brothers, E N., Kudin, K N., Staroverov, V N., Keith, T A., Kobayashi, R., Normand, J., Raghavachari, K., Rendell, A P., Burant, J C., Iyengar, S S., Tomasi, J., Cossi, M., Millam, J M., Klene, M., Adamo, C., Cammi, R., Ochterski, J W., Martin, R L., Morokuma, K., Farkas, O., Foresman, J B. & Fox, D J, Gaussian 16 rev. C.01, (2016).

11. E. D. Glendening, J, K. Badenhoop, A. E. Reed, J. E. Carpenter, J. A. Bohmann, C. M. Morales, P. Karafiloglou, C. R. Landis, F. Weinhold, NBO 7.0 (2018).
12. Maeda, S., Ohno, K. & Morokuma, K. Systematic exploration of the mechanism of chemical reactions: The global reaction route mapping (GRRM) strategy using the addf and AFIR methods. *Phys. Chem. Chem. Phys.* **15,** 3683-3701 (2013).
13. Software to optimize reaction paths along the user's expected ones, HPC systems inc., http://www.hpc.co.jp/chem/react2.html (written in japanese).
14. Berne, B J., Ciccotti, G. & Coker, D F. *Classical and quantum dynamics in condensed phase simulations: Proceedings of the international school of physics*. World Scientific, 1998.
15. Henkelman, G. & Jónsson, H. Improved tangent estimate in the nudged elastic band method for finding minimum energy paths and saddle points. *J. Chem. Phys.* **113,** 9978-9985 (2000).
16. Hanwell, M D., Curtis, D E., Lonie, D C., Vandermeersch, T., Zurek, E. & Hutchison, G R. Avogadro: An advanced semantic chemical editor, visualization, and analysis platform. *J. Cheminform.* **4,** 17 (2012).
17. Persistence of Vision Pty. Ltd. Persistence of Vision Raytracer (version 3.7) retrieved from http://www.povray.org/download/.
18. Neese, F. The ORCA program system. *WIREs Comput. Mol. Sci.* **2,** 73-78 (2011).
19. Neese, F. Software update: The ORCA program system–version 5.0. *WIREs Comput. Mol. Sci.* **12,** (2022).
20. Knizia, G. Intrinsic atomic orbitals: An unbiased bridge between quantum theory and chemical concepts. *J. Chem. Theory Comput.* **9,** 4834-4843 (2013).
21. Knizia, G. & Klein, J E. Electron flow in reaction mechanisms--revealed from first principles. *Angew. Chem. Int. Ed.* **54,** 5518-5522 (2015).
